# Supplementary material for: Allosteric regulation and crystallographic fragment screening of SARS-CoV-2 NSP15 endoribonuclease
Source: Nucleic Acids Res. 2023 Apr 28;51(10):5255–70. doi: 10.1093/nar/gkad314 (PMC10250223; doi:10.1093/nar/gkad314)
Supplement: gkad314_Supplemental_Files [file gkad314_supplemental_files.zip › Supplementary datasets.zip!/224/Refine_9/01-BUSTER/Cycle-2/LIST.html]

Refining

**BUSTER 2.X**: A suite of programs for macromolecular structure refinement by maximum-likelihood and Bayesian methods  
Copyright © 1992-2020 Global Phasing Ltd.  
All rights reserved.  
Authors: G. Bricogne, E. Blanc, M. Brandl, C. Flensburg, P. Keller, W. Paciorek, P. Roversi, O. Smart, C. Vonrhein, T. Womack  

Please reference use of the software as:

- BUSTER-TNT 2.X, Global Phasing Ltd, Sheraton House, Cambridge CB3 0AX, UK

Version: 2.10.3 for linux64-ifort made on May 20 2020 at 01:14:34  
Licenced to: Diamond Light Source, Scientific Software  

```
---


### Parsing of the BUSTER cardfile

TITLE : autoBUSTER refinement

   Cell information

   Symmetry information

 Using CCP4 X-ray form factors.

   Missing structure chemical composition

 Using ATOM block for chemical composition.


   Shell information

### Parsing of the TNT cardfile


---


### Reading observations file

 Number of reflexions in input:  58343

      Number of accepted reflexions:  52749

      Number of rejected reflexions:      0

   Reflexions in test set:            2702

   Reflexions in working set:        50047


---


### 1. Generating initial model

 2.10 * Shannon sampling rate. NX NY NZ:   288  288  240


  Effective Shannon rates are:    2.29    2.29    2.55


   Allocated BUFFER of size:     76.465 Mb.

   Initial Geometry Information


 No missing atoms declared


   Summary of model volumes

 No missing atoms declared: no prior computed


 Computing bulk solvent model scattering


 NCSMASK PDB radius for solvent mask:     2.15 Angstrom

 running PDBSET (setting cell and spacegroup P1) ... done
 running NCSMASK [1] (creating mask, SMOOTH 1.04842) ... done
 running MAPDUMP (to check if reduction to unit cell is needed) ... done
 running MAPMASK (converting mask to map) ... done

   Generation of babslv by theta-filter

   Electron density in frg mask:  0.420 Å-1

 FOBS  put on rough absolute scale by Wilson plot :
           K        =  0.64699E+02
           B        =     -7.265

 

### Cycle 0001: MaxLik scaling and refinement


   MaxLik Scaling Cycle 0001

   Wilson plot

   Statistics in resolution bins just after scaling
```

|  |  |
| --- | --- |
|  | - Rxpct factors plot- Rxpct factors table- <Log-Lik Gain>- Ln Sigma A- Correlation Coefficients |

```
   <Log-Likelihood> of null-hypothesis

Structure Factor Amplitudes


   Average Figure of Merit

 Switching off refinement of overall temperature factor


 Added B =    -0.0166 Å2 to all atoms in fragment


 Added B =    -0.0166 Å2 to B_SOLV

```
gelly refinement module
 Authors (2004-2009) O. S. Smart, C. Flensburg, W. Paciorek, C. Vonrhein, 
                     M. Brandl, T. O. Womack and G. Bricogne
         (2010-2011) O. S. Smart, C. Flensburg, W. Paciorek, C. Vonrhein, 
                     T. O. Womack and G. Bricogne
         (2012-2014) O. S. Smart, C. Flensburg, W. Paciorek, C. Vonrhein, 
                     and G. Bricogne
         (2015-2020) G. Bricogne, C. Flensburg, W. Paciorek, C. Vonrhein
 Copyright © 2004-2020 Global Phasing Ltd. 
 All rights reserved.
 gelly module compilation date May 20 2020 01:11:20
 
 gelly uses routines from TNT geometry. References: 
     Tronrud, D. E., Ten Eyck, L. F., Matthews, B. W. (1987)
     "An Efficient General-Purpose Least-Squares Refinement Program 
     for Macromolecular Structures". Acta Crystallogr A, 43:489-501.
     Tronrud, D. E. (1997). "The TNT Refinement Package", 
     in Macromolecular Crystallography, Part B, Eds Charlie Carter 
     and Robert Sweet, Methods in Enzymology, 277:306-319.
 wildcard matching function by Julian Robichaux http://www.nsftools.com

 No -random_seed flag found. Random number stream initialised using:
2147483562
2147483398

 Control flags set to:  -autoncs -hydrogens_input_occ2zero \
                     -hydrogens_output_occ2heavy -screen \
                     100 -screen_sigma \
                     3.0 -keeppdb \
                     1000000 -taomonitor_verbose \
                     2 
 

 ############### Starting setup via TNT routines

 Setting up tnt routines including license check:

                   TNT Stereochemistry Program
 Version: 5.22.3.0
 Licence for Diamond Light Source, Scientific Software is valid
 
 Using TNT geometry routines to load the tnt control file: gelly.tnt
 Using TNT routines to load initial coordinates from file fragment.cor
 Expecting 6506 atoms

 Found     2197 "NOTE BUSTER_RESIDUE_ATOM_DATA" cards
 Have copied information for    6506 atoms from TNT to gelly's store 
 
 The TNT WEIGHT cards used by this run:
 -----WEIGHT       BOND     2.0
 -----WEIGHT       ANGLE    2.0
 -----WEIGHT       IMPROPER 0.0
 -----WEIGHT       TORSION  2.0
 -----WEIGHT       PSEUDO   0.0
 -----WEIGHT       TRIGONAL 2.0
 -----WEIGHT       PLANE    5.0
 -----WEIGHT       CONTACT  5.0
 -----WEIGHT       BCORREL  10.0
 -----WEIGHT       CHIRAL   5.0
 -----WEIGHT       NCS      50.0
 -----WEIGHT       IDEAL    4.0
 -----WEIGHT       RFACTOR  2.29
 
 Dictionary versions picked up from NOTE BUSTER_RESTRAINT_DICT cards as: 
 ------ protgeo_eh99.dat (V1.6.4.17) 20191001  STANDARD AMINO ACID DICTIONARY. BONDS AND ANGLES FROM ENGH AND HUBER EH99. OTHER VALUES BASED ON PREVIOUS TNT OR TAKEN FROM CCP4. INCLUDES HYDROGEN ATOMS.
 ------ exoticaa.dat (V1.3.4.15) 20200327  COLLECTION OF NON-STANDARD AMINO ACIDS, MAINLY EH91 WITHOUT IDEAL DISTANCE INFO
 ------ nuclgeo.dat (V1.13.4.8) 20190909
 ------ bcorrel.dat (V1.15.10.2) 20190616
 ------ contact.dat (V1.15.12.10) 20191121
 ------ idealdist_contact.dat (V1.3.4.12) 20191001  IDEAL-DISTANCE CONTACT TERM DATA AS USED IN PROLSQ. VALUES USED HERE ARE BASED ON DATA HARVESTING OF PDB AS OF 20190109.
 ------ restraints for LIG (Unknown) from cif dictionary Z927400026.cif
 ------ restraints for CIT (CITRIC ACID) from cif dictionary CIT.cif; generated by GRADE_PDB_LIGAND 1.2.12 (October 4 2016) using MOGUL 1.7.2(RC1), CSD as538be, with quantum mechanics RM1 ((DatabaseID PDB-CIT))
 ------ assume.dat (V1.9.4.2) 20180705
 
 Have picked up spacegroup name for pdb output as 'P 63      '
 Setting up Chain/Residue arrays
 
 Have command line specification -autoncs*
     Have found       1 NCS relations
     Pairwise matching residue number and type between chains: 
                    A        B        S        W    
          A         .       99%       -        -   
          B                  .        -        -   
          S                           .        -   
          W                                    .   
       Table key--                                                       
          all   : two chains have 100% match and same number of residues 
         multi  : ditto and they contribute to a multi chain ncs cluster 
          ***%  : two chains have >80% matching residue number/type      
           -    : chains appear to be unrelated                          
      chain A     have identified ncs to     1 other chains. So will weight each autoncs by (       6.666/#)=      6.6660
      chain B     have identified ncs to     1 other chains. So will weight each autoncs by (       6.666/#)=      6.6660
      chain S     have identified ncs to     0 other chains. 
      chain W     have identified ncs to     0 other chains. 
     For .Gelly cards produced see below - after pruning
 
 Setting up BUSTER_SET groups
 Have found    89 BUSTER_SET cards
 Now silently applying automatic/dictionary set definitions. For a verbose listing use '-verbose_set' flag.
 Applying user defined set definitions:
 =====NOTE BUSTER_SET ncsautoXcld = Water --> set 'ncsautoXcld' now has   966 atoms 
 =====NOTE BUSTER_SET ncsautoABset = Chain_A \ ncsautoXcld --> set 'ncsautoABset' now has  2770 atoms 
 Have defined    49 sets: 
      set:                 'All' has   6506 atoms in   1666 residues - automatically defined
      set:               'Empty' has      0 atoms in      0 residues - automatically defined
      set:            'ExoticAA' has      0 atoms in      0 residues - defined by dictionary file
      set:           'ExoticNUC' has      0 atoms in      0 residues - defined by dictionary file
      set:              'NotHET' has   5514 atoms in    698 residues - defined by dictionary file
      set:             'Chain_A' has   2770 atoms in    350 residues - automatically defined
      set:             'Chain_B' has   2770 atoms in    350 residues - automatically defined
      set:             'Chain_S' has    567 atoms in    567 residues - automatically defined
      set:             'Chain_W' has    399 atoms in    399 residues - automatically defined
      set:               'Water' has    966 atoms in    966 residues - defined by dictionary file
      set:          'StdProtein' has   5514 atoms in    698 residues - defined by dictionary file
      set:             'Protein' has   5514 atoms in    698 residues - defined by dictionary file
      set:                'Back' has   2794 atoms in    698 residues - defined by dictionary file
      set:                'Side' has   2720 atoms in    650 residues - defined by dictionary file
      set:        'RnaCanonical' has      0 atoms in      0 residues - defined by dictionary file
      set:        'DnaCanonical' has      0 atoms in      0 residues - defined by dictionary file
      set:     'RnaDnaCanonical' has      0 atoms in      0 residues - defined by dictionary file
      set:     'DnaRnaCanonical' has      0 atoms in      0 residues - defined by dictionary file
      set:         'RnaExtended' has      0 atoms in      0 residues - defined by dictionary file
      set:         'DnaExtended' has      0 atoms in      0 residues - defined by dictionary file
      set:      'RnaDnaExtended' has      0 atoms in      0 residues - defined by dictionary file
      set:      'DnaRnaExtended' has      0 atoms in      0 residues - defined by dictionary file
      set:                 'Dna' has      0 atoms in      0 residues - defined by dictionary file
      set:                 'Rna' has      0 atoms in      0 residues - defined by dictionary file
      set:              'RnaDna' has      0 atoms in      0 residues - defined by dictionary file
      set:              'DnaRna' has      0 atoms in      0 residues - defined by dictionary file
      set:             'Nucleic' has      0 atoms in      0 residues - defined by dictionary file
      set:             'Polymer' has   5514 atoms in    698 residues - defined by dictionary file
      set: 'BufferWeichenberger' has     13 atoms in      1 residues - defined by dictionary file
      set:    'BufferAdditional' has      0 atoms in      0 residues - defined by dictionary file
      set:              'Buffer' has     13 atoms in      1 residues - defined by dictionary file
      set:         'MetalAlkali' has      0 atoms in      0 residues - defined by dictionary file
      set:  'MetalAlkalineEarth' has      0 atoms in      0 residues - defined by dictionary file
      set:     'MetalTransition' has      0 atoms in      0 residues - defined by dictionary file
      set: 'MetalPostTransition' has      0 atoms in      0 residues - defined by dictionary file
      set:    'MetalLanthanides' has      0 atoms in      0 residues - defined by dictionary file
      set:               'Metal' has      0 atoms in      0 residues - defined by dictionary file
      set:         'SugarAlphaD' has      0 atoms in      0 residues - defined by dictionary file
      set:         'SugarAlphaL' has      0 atoms in      0 residues - defined by dictionary file
      set:               'Sugar' has      0 atoms in      0 residues - defined by dictionary file
      set:               'Other' has     26 atoms in      2 residues - defined by dictionary file
      set:            'Hydrogen' has      0 atoms in      0 residues - defined by dictionary file
      set:         'nonHydrogen' has   6506 atoms in   1666 residues - defined by dictionary file
      set:             'OccZero' has      0 atoms in      0 residues - defined by dictionary file
      set:          'OccNonZero' has   6506 atoms in   1666 residues - defined by dictionary file
      set:             'OccFull' has   6461 atoms in   1663 residues - defined by dictionary file
      set:          'OccNonFull' has     45 atoms in      3 residues - defined by dictionary file
      set:         'ncsautoXcld' has    966 atoms in    966 residues - user specified
      set:        'ncsautoABset' has   2770 atoms in    350 residues - user specified
 No command line arguments '-occ_allhet' or '-occ_restyp' so will not setup automated occcupancy refinement
 
 Interpreting CONSTANT and FREE cards: 
 =====CONSTANT     OCC
 Have found and interpreted      1 CONSTANT cards in the TNT input. 
 Before any COMBINE - Number of atoms with fixed XYZ =     0
 Before any COMBINE - Number of atoms with fixed B   =     0
 Before any COMBINE - Number of atoms with fixed OCC =  6506
 Before any COMBINE - Number of atoms with fixed BIJ =   966
                            cf total number of atoms =  6506
 
 There are no COMBINE cards to be found in the TNT card input 
 
 There are no NOTE BUSTER_COMBINE_RES cards to be found in the TNT card input 

 ############### Working out full connectivity list


 ############### Working out equivalent atoms


 Residue with most atoms given below:
        A|58:N(TRP)         
        A|58:CA(TRP)        
        A|58:CB(TRP)        
        A|58:CG(TRP)        
        A|58:CD1(TRP)       
        A|58:NE1(TRP)       
        A|58:CE2(TRP)       
        A|58:CD2(TRP)       
        A|58:CE3(TRP)       
        A|58:CZ3(TRP)       
        A|58:CH2(TRP)       
        A|58:CZ2(TRP)       
        A|58:C(TRP)         
        A|58:O(TRP)         


 Have no '-equiv_check_plus' argument - so will take atom type into
 account when checking for equivalence of otherwise identical atoms.
 

 ############### Looking at dihedrals


 ############### Looking at chirals

 NOTE BUSTER_CHIRAL_ANGLECUT card found - read value=    -31.00 degs from it
 NOTE BUSTER_CHIRAL_SIGMA card found - read value=      1.70 degs from it
 RANGE allowed for B variables in refinement:   from      3.00000 to    300.00000

 ############### Preparing for PDB output


 ############### Processing extra restraints explicitly defined

 No 'NOTE BUSTER_TARGET' cards or -target?? args found (used to read coordinates files for soft ncs to external)
 No 'NOTE BUSTER_OCCSUM'     cards found - so not using sum of occupancy restraints
 No 'NOTE BUSTER_DISTANCE'   cards found - so not setting up any additional atom-atom distance restraints
 No 'NOTE BUSTER_UTILANGLE'  cards found - so not setting up any additional restraints on any angle
 No 'NOTE BUSTER_UTILTOR'    cards found - so not setting up any additional sinusoidal restraints on torsions
 
 --- Have some similarity definitions 
     Similarity restraints on RMSD use superposition routines from: 
     Coutsias, E.A., Seok, C., Dill, K.A.(2004)
     "Using quaternions to calculate RMSD", J. Comput. Chem., 25:1849-1857 

 Have found    1 'NOTE BUSTER_SIM_DEFINE' cards 
     Maximum number of chains involved in any SIM group (excluding the template)=     1


 ########## Interpreting card: NOTE BUSTER_SIM_DEFINE ncsautoAB ncsautoABset B
     Number of atoms in template set=  2770
     Number of related chains (other than template) in SIM set=    1
     List of chains to be restrained (excluding template)=   B   
     Pairwise RMSD, XYZ in Angstroms, for SIM group: ncsautoAB
                 A         B   
     A         -----     ----- 
     B         0.653     ----- 
     Overall RMSD,XYZ for the SIM group is     0.653 Angstroms
     Pairwise rms deviation of [individual B's minus their set/chain average] in Angs**2
                 A         B         Bavg
     A         -----     -----     86.90
     B         27.73     -----     68.02
     Overall RMSD,B   for the SIM group is    27.725 Angs**2
     The number of common atoms for the above fits:
                 A         B   
     A         -----     ----- 
     B          2757     ----- 
     (compared to the template set that has    2770 atoms in it)
     List of atoms that are absent in one or more chains for this group: 
                                    A    B   
                    C1      401     +    -
                    O1      401     +    -
                    O2      401     +    -
                    C2      401     +    -
                    C3      401     +    -
                    O7      401     +    -
                    C4      401     +    -
                    C5      401     +    -
                    O3      401     +    -
                    O4      401     +    -
                    C6      401     +    -
                    O5      401     +    -
                    O6      401     +    -
     (key  +  atom present for this chain 
           -  atom absent  for this chain)
 transforms for each of the superpositions orthogonal matrix r11 r12 r13 r21 r22 r23 r31 r32 r33 tx ty tz (PDBSET syntax): 
TRANS_AB -0.877148 -0.480058 -0.012516 -0.480080  0.877224 -0.001364  0.011634  0.004812 -0.999921    33.905    33.021    15.299
 reverse transforms 
TRANS_BA -0.877148 -0.480080  0.011634 -0.480058  0.877224  0.004812 -0.012516 -0.001364 -0.999921    45.414   -12.765    15.767
 
 Setting up LSSR interatomic distance restraints
 Interpreting card: NOTE BUSTER_SIM_RESTRAIN_LSSR ncsautoAB 6.666
        setting up interatomic distance pair restraints with:
        Dmin=         0.00
        Dmax=         5.50
        sigma=        0.20
        Vmax=         3.00
        weight=       6.67
        number of distance pairs defined for this set is      35121
 Have not supplied either '-sim_swap_equiv' or '-sim_swap_equiv_plus' argument 
   so will not try swaping equivalent atoms to improve NCS and/or target agreement.
 
 NCS by LSSR outlier analysis
 -autoncs option automatic NCS prune routine -------- 
 Residues pruned out by gradient condition:
          A|253     B|253
  
 Residues pruned out by function/max condition:
          A|149     A|150     A|173     A|187     A|188     A|216     A|220 
          A|259     A|346    A|ENDA     B|149     B|150     B|173     B|187 
          B|188     B|216     B|220     B|259     B|346    B|ENDA 
  
 Total number of residues for which LSSR restraints have been pruned out is       22 out of      698 participating
 Number of LSSR restraints pruned out is      1456 this is    4.1% of the total           35121
  
 -autoncs .Gelly cards suitable for adaptation for manual definition/pruning
           (note auto pruning is done in a slightly different way so exact results may differ slightly)
 -autoncs option .Gelly cards also written to file  auto_gelly_cards.txt
NOTE BUSTER_SET ncsautoXcld = Water    # card added - produced by -autoncs
NOTE BUSTER_SET ncsautoXcld = ncsautoXcld + { A|149 A|150 A|173 A|187 A|188 }    # card added - produced by -autoncs
NOTE BUSTER_SET ncsautoXcld = ncsautoXcld + { A|216 A|220 A|253 A|259 A|346 }    # card added - produced by -autoncs
NOTE BUSTER_SET ncsautoXcld = ncsautoXcld + { A|ENDA }    # card added - produced by -autoncs
NOTE BUSTER_SET ncsautoABset = Chain_A \ ncsautoXcld    # card added - produced by -autoncs
NOTE BUSTER_SIM_DEFINE ncsautoAB ncsautoABset B    # card added - produced by -autoncs
NOTE BUSTER_SIM_RESTRAIN_LSSR ncsautoAB 6.666    # card added - produced by -autoncs

 LSSR function contribution before pruning    119648.3
                             after pruning    104483.8
 Graph of which residues pruned from LSSR: ncs_pruned.mtv
 Graph of LSSR function outliers ncs_function_outliers.mtv
 Graph of LSSR gradient outliers ncs_gradient_outliers.mtv
 LSSR - analysis of distance difference distribution for NCS
         DeltaD less than 0.5 Angs:  proportion of population= 96.80% average DeltaD of subset     0.096 Angs
 Histogram of LSSR deltaD distribution ncs_histogram.mtv
 
 Interpreting card: NOTE BUSTER_TLS_SET tls1 { A|* }
 Interpreting card: NOTE BUSTER_TLS_SET tls2 { B|* }
 Interpreting card: NOTE BUSTER_TLS_SET tls1 { A|* }
 Interpreting card: NOTE BUSTER_TLS_T   tls1     -5.14088E-02  1.73416E-02 -8.39463E-02 -8.98636E-03  2.71161E-02 -1.44550E-02
 Interpreting card: NOTE BUSTER_TLS_L   tls1      5.75955E-01  6.03476E-01  7.20382E-02  4.29501E-01 -2.01688E-01 -7.44442E-02
 Interpreting card: NOTE BUSTER_TLS_S   tls1      2.27569E-01 -1.40005E-01  1.08103E-01 -1.53415E-01 -6.25968E-02 -9.66768E-02 -5.90336E-02  8.69448E-04
 Interpreting card: NOTE BUSTER_TLS_O   tls1     -2.34016E+01 -8.37990E+01  1.17779E+01
 Interpreting card: NOTE BUSTER_TLS_SET tls2 { B|* }
 Interpreting card: NOTE BUSTER_TLS_T   tls2     -6.53873E-02 -2.61952E-02 -1.61508E-02  1.87470E-02  4.10405E-02  3.19663E-03
 Interpreting card: NOTE BUSTER_TLS_L   tls2      3.60326E-01  5.33835E-01  2.67053E-01  2.19756E-01 -1.98637E-01 -2.16915E-01
 Interpreting card: NOTE BUSTER_TLS_S   tls2     -1.51205E-02  2.91585E-02 -3.20999E-02 -6.51209E-03  1.30308E-01  8.58995E-03 -4.97491E-02  3.47416E-02
 Interpreting card: NOTE BUSTER_TLS_O   tls2      2.21707E+01 -7.20650E+01  3.37962E+00
 Interpreting card: NOTE BUSTER_TLS_FIX tls1 ALL
 Interpreting card: NOTE BUSTER_TLS_FIX tls2 ALL


 TLS information. Total number of groups:     2


 Group   Natoms    FixRB   FixTLS   Group Tag    Group Spec
     1     2770      Y        Y     tls1    { A|* }
     2     2770      Y        Y     tls2    { B|* }
 
NOTE BUSTER_TLS_SET tls1 { A|* }
NOTE BUSTER_TLS_T   tls1     -5.14088E-02  1.73416E-02 -8.39463E-02 -8.98636E-03  2.71161E-02 -1.44550E-02
NOTE BUSTER_TLS_L   tls1      5.75955E-01  6.03476E-01  7.20382E-02  4.29501E-01 -2.01688E-01 -7.44442E-02
NOTE BUSTER_TLS_S   tls1      2.27569E-01 -1.40005E-01  1.08103E-01 -1.53415E-01 -6.25968E-02 -9.66768E-02 -5.90336E-02  8.69448E-04
NOTE BUSTER_TLS_O   tls1     -2.34016E+01 -8.37990E+01  1.17779E+01
NOTE BUSTER_TLS_FIX tls1 ALL
 
NOTE BUSTER_TLS_SET tls2 { B|* }
NOTE BUSTER_TLS_T   tls2     -6.53873E-02 -2.61952E-02 -1.61508E-02  1.87470E-02  4.10405E-02  3.19663E-03
NOTE BUSTER_TLS_L   tls2      3.60326E-01  5.33835E-01  2.67053E-01  2.19756E-01 -1.98637E-01 -2.16915E-01
NOTE BUSTER_TLS_S   tls2     -1.51205E-02  2.91585E-02 -3.20999E-02 -6.51209E-03  1.30308E-01  8.58995E-03 -4.97491E-02  3.47416E-02
NOTE BUSTER_TLS_O   tls2      2.21707E+01 -7.20650E+01  3.37962E+00
NOTE BUSTER_TLS_FIX tls2 ALL


 TLS: mmCIF group specification begin


#
loop_
_pdbx_refine_tls_group.id
_pdbx_refine_tls_group.refine_tls_id
_pdbx_refine_tls_group.beg_PDB_ins_code
_pdbx_refine_tls_group.beg_auth_asym_id
_pdbx_refine_tls_group.beg_auth_seq_id
_pdbx_refine_tls_group.end_PDB_ins_code
_pdbx_refine_tls_group.end_auth_asym_id
_pdbx_refine_tls_group.end_auth_seq_id
_pdbx_refine_tls_group.selection
_pdbx_refine_tls_group.selection_details
   1   1 . A   -1 . A  346 . '{ A|* }'
   2   1 . A  401 . A  401 . '{ A|* }'
   3   2 . B   -1 . B  346 . '{ B|* }'
   4   2 . B  401 . B  401 . '{ B|* }'
#


 TLS: mmCIF group specification end


 Interpreting card: NOTE BUSTER_ANISO_SET Empty
 Interpreting card: NOTE BUSTER_ANISO_WEIGHT SCA  16.0
 Interpreting card: NOTE BUSTER_ANISO_WEIGHT SPH   0.6    0.6
 Interpreting card: NOTE BUSTER_ANISO_WEIGHT SIM   7.8    4.8    2.6    0
 Interpreting card: NOTE BUSTER_ANISO_WEIGHT RIG  21.5   21.5    3.7    0


 After TLS and ANISO: Number of atoms with fixed/combined xyz =     0
 After TLS and ANISO: Number of atoms with fixed/combined B   =     0
 After TLS and ANISO: Number of atoms with fixed/combined OCC =  6506
 After TLS and ANISO: Number of atoms with fixed/combined BIJ =  6506
 After TLS and ANISO: Number of atoms in a TLS group          =  5540
                                     cf total number of atoms =  6506
 

 ############### Setup state vector

 No free_vect_ulim_xyz argument found: using default of  100.000
 No free_vect_llim_xyz argument found: using default of -100.000
 Number of parameters added for TLS description                 =       0
 Number of parameters to describe molecular conformation        =   26024

 ############### Setup free vector

 Number of free parameters to be adjusted in refinement   =   26024

 ############### Setting up own geometry


 No command line argument '-tnt_fn' so will use gelly to do geometry function calculations

 ============= Call TNT functions


 ============= Get TNT symmetry information

 
      Getting symmetry operators from TNT. 
          gelly will classify symmetry using pdb-like convention: 
               SYMOP   SYMMETRY
              NNNMMM   OPERATOR
                1555   X,Y,Z
                2555   -Y,X-Y,Z
                3555   -X+Y,-X,Z
                4555   -X,-Y,1/2+Z
                5555   Y,-X+Y,1/2+Z
                6555   X-Y,X,1/2+Z
          where NNN -> operator number and MMM -> translation vector
 

 ============= Own Gelly geometry/restraints setup


 ----------- Identify bond-types


    Setting up GEOMBONDREC_TYPE_HASH* to be able to map a given bond type (e.g.
    "single") to an internal integer (GBOND_TYPE=1) etc.

    Using default  GEOMBONDREC_TYPE_HASH_STR='SING* 1 DOUB* 2 TRIP* 3 AROM* 4 DELO* 4 METAL* 5':
    to override default use a NOTE BUSTER_GEOMBONDREC_TYPE_HASH_STR gelly card.

 ----------- Classify torsion into peptide omega and other


 ----------- Setup of non-bonded terms

 Ideal-distance contact term setup:
    Restraint function constants (normally these values are from NOTE BUSTER_IDEAL_CONTACT_* cards)
    Sigma for general contacts      0.200 Angs
    Sigma for 1-4 contacts          0.100 Angs (contacts across a torsion)
    Sigma for 1-5 contacts          0.150 Angs
    Sigma for 1-N contacts          0.200 Angs
    Sigma for HBOND pairs           0.100 Angs (contacts between HB_types A and D or B's)
    Contact distance increment for each atom in HBOND          -0.150 Angs 
    Contact distance increment for H-bond acceptor              0.100 Angs 
    Contact distance increment for 1-4 with an O atom          -0.050 Angs 
    Contact distance increment for 1-4 with an N atom          -0.180 Angs 
    Contact distance increment for 1-4 with an C atom          -0.370 Angs 
    Contact distance increment for 1-4 with an S atom          -0.150 Angs 
    Contact distance increment for 1-4 with an P atom          -0.150 Angs 
    Contact distance increment for 1-4 with any other atom     -0.150 Angs 
    All atoms matched for CCP4 atom type.
    Number of CCP4 atom types used is         25 (H-bound and not H-bound counted separately)

 Setting up short/ideal-distance contacts
   Using gelly special position handling. (For TNT instead rerun with argument '-special_tnt')

   No command line argument '-special_all' specified. We will auto-generate
   EXCLUDE cards only for atom-atom contacts across symmetry (i.e. on special
   position) when this involves a contact within a residue to itself.

   Have set up non-bonded exclusions - 1-3 contacts
   Atom A|27:CA         makes the most 1-3 contacts:  6

   Have set up non-bonded exclusions - 1-4 contacts
   Atom A|27:N          makes the most 1-4 contacts:  7

   Have set up non-bonded exclusions - 1-5 contacts
   Atom A|28:C.A        makes the most 1-5 contacts: 10

 ############################################################################################################
 ###################################################### setting up contact terms
 ############################################################################################################


 ============= Call Gelly versions of TNT functions

 WARNING: 1-4 contact without 1-(2,3)-4 data for A|401:O2(CIT)        and A|401:C3(CIT)        X-X
 WARNING: 1-4 contact without 1-(2,3)-4 data for A|401:C2(CIT)        and A|401:O6(CIT)        X-X
 WARNING: 1-4 contact without 1-(2,3)-4 data for B|401:C01(LIG)       and B|401:N05(LIG)       X-X
 WARNING: 1-4 contact without 1-(2,3)-4 data for B|401:N08(LIG)       and B|401:O11(LIG)       X-X
 WARNING: 1-4 contact without 1-(2,3)-4 data for B|401:C09(LIG)       and B|401:C12(LIG)       X-X
 WARNING: 1-4 contact without 1-(2,3)-4 data for B|401:C10(LIG)       and B|401:C13(LIG)       X-X

       TERM      weight      number      TNT_fn    Gelly_fn        diff
       BOND       2.000        5655    5161.365    5161.365       0.000
       ANGL       2.000        7674   11703.798   11703.798       0.000
       IMPR       0.000           0       0.000       0.000       0.000
       TORS       2.000        1968    2797.950    2797.950      -0.000
       TRIG       2.000           0       0.000       0.000       0.000
       PLAN       5.000         963   14444.369   14444.369       0.000
       CONT       5.000           0      92.663       0.000      92.663
       BCOR      10.000        5630  167870.799  171693.888   -3823.089


 ============= Call Gelly geometry functions

   Switching torsion functional form to sinusoidal rather than split harmonic functional.
      if you want this not to happen then rerun with '-torsharm'
      Weight for sinusoidal torsion=     2.000
   BCOR function/gradient will be split between H and non-H atoms (as for any atom pair). Use '-bcorrel_all_in_hydrogens' to change this.

 ----------- Fix B-correlation restraints

 
 Checking for bonds that do not have a bcorrel restraint defined
 We will wipe all existing BCORrels if OCC=0.0 atom(s) is/are involved. This can be' switched off via the '-bcorrel_nowipe_zeroocc' flag.
 We will wipe all existing BCORrels if only non-hydrogen atom(s) with fixed B(iso) are involved. This can be switched off via the '-bcorrel_nowipe_fixb_nonhydrogens' flag.
 We will keep all existing BCORrels if hydrogen atom(s) with fixed B(iso) is/are involved. This can be switched off via the '-bcorrel_wipe_fixb_hydrogens' flag.
 No -bcorrel_missing_sigma found. So will set sigma to     2.5 Ang**2 for any bond with no existing BCORrel
 No -bcorrel_missing_sigma_hydrogens found. So will set sigma to     1.0 Ang**2 for any bond with no existing BCORrel involving hydrogens

 No active BCORels found involving OCC=0.0 atom(s)


 No active BCORels found involving fixed-B (only) non-hydrogen atom(s)

    add a default BCOR for bond A|401:C1(CIT) A|401:C2(CIT)
    add a default BCOR for bond A|401:C1(CIT) A|401:O1(CIT)
    add a default BCOR for bond A|401:C1(CIT) A|401:O2(CIT)
    add a default BCOR for bond A|401:C2(CIT) A|401:C3(CIT)
    add a default BCOR for bond A|401:C3(CIT) A|401:C4(CIT)
    add a default BCOR for bond A|401:C3(CIT) A|401:C6(CIT)
    add a default BCOR for bond A|401:C3(CIT) A|401:O7(CIT)
    add a default BCOR for bond A|401:C4(CIT) A|401:C5(CIT)
    add a default BCOR for bond A|401:C5(CIT) A|401:O3(CIT)
    add a default BCOR for bond A|401:C5(CIT) A|401:O4(CIT)
    add a default BCOR for bond A|401:C6(CIT) A|401:O5(CIT)
    add a default BCOR for bond A|401:C6(CIT) A|401:O6(CIT)
    add a default BCOR for bond B|401:C01(LIG) B|401:C02(LIG)
    add a default BCOR for bond B|401:C02(LIG) B|401:C03(LIG)
    add a default BCOR for bond B|401:C02(LIG) B|401:C04(LIG)
    add a default BCOR for bond B|401:C04(LIG) B|401:N05(LIG)
    add a default BCOR for bond B|401:C04(LIG) B|401:C06(LIG)
    add a default BCOR for bond B|401:C06(LIG) B|401:O07(LIG)
    add a default BCOR for bond B|401:C06(LIG) B|401:N08(LIG)
    add a default BCOR for bond B|401:N08(LIG) B|401:C09(LIG)
    add a default BCOR for bond B|401:N08(LIG) B|401:C13(LIG)
    add a default BCOR for bond B|401:C09(LIG) B|401:C10(LIG)
    add a default BCOR for bond B|401:C10(LIG) B|401:O11(LIG)
    add a default BCOR for bond B|401:O11(LIG) B|401:C12(LIG)
    add a default BCOR for bond B|401:C12(LIG) B|401:C13(LIG)

 Have added a total of        25 BCORrels for bonds which do not already have a BCORrel


 No bonds (for potential BCORrel addition) were skipped due to both atoms having constant/fixed B(iso)


 ----------- Deactivate hydrogen-free planes if hydrogen atoms present

 A total of       0 planes have been deactivated because they are duplicated hydrogen-free and with-hydrogen forms.

 ----------- Handle peptide omega angle(s)

      Torsion angles split into peptide omega and other
         Number of peptide omega angles is       698
         Number of other torsion is             1270
      Peptide omega angles are monitored rather than directly restrained (PLANE used instead).

 ----------- Fix cis-PRO

 Now apply the fix to get correct restraints for cis PRO residues
   Have got      11 bonds records and      26 angles
   from GEOMETRY cards for the special PROC residue
   Weight for ideal-distance contact term=     4.000

 ----------- Prepare SSBOND/LINK for output PDB file


 ----------- Prepare for QM


 ----------- Prepare symmetry in utility distance restraints


 ############### Setting up Rhofit contacts (if needed)

 No ligand-(fixed protein) contact term will be used

 ############### Working out initial function and gradient


   MaxLik Scaling Cycle 0002
 
 Initial functional value = 4.607042E+05 rms gradient=      715.856
 X-ray contribution to function value = 1.246057E+05
 Geometry contribution (inc ncs)      = 3.360985E+05
 Geometry function summary
     term                           number     weight       rms        GooF      function     fn/numb
     ================================================================================================
     BOND bond lengths (angs)       5655       2.000      0.0136       0.676      5161.37       0.913
     ANGL bond angles (degs)        7674       2.000        1.47       0.873     11703.80       1.525
     TORS split-harmonic tors       1270       0.000       16.68       1.050         0.00       0.000
     SINTOR sinusoidal tors         1270       2.000       16.68                  2196.42       1.729
     PLAN general plane (angs)       963       5.000      0.0346       1.732     14444.37      14.999
     BCORrelations (angs^2)         5655      10.000       4.946       1.765    176200.98      31.158
     CONT (bad contacts)               0       5.000                                 0.00
     IDEAL(ideal-dist contact)      5337       4.000                             21899.74
     CHIRAL (gelly semiharmon)       740       5.000                                 8.07
     SIM similarity restraint for NCS and/or TARGET                             104483.81
     OCCSUM restraint on sum of occupanies                                           0.00       as none defined
     DISTAN utility restraints interatomic distance                                  0.00       as none defined
     UTILANGLE utility angle restraints                                              0.00       as none defined
     UTILTOR utility restraints on torsion angles                                    0.00       as none defined
     Peptide omega torsion angles                           7.50 degs - monitor only
 
 WARNING: Have |delta/sigma| deviations > 5.0 sigma. Number of outliers for each term: 
 WARNING:         2 bond angles.  Worst is     6.2 sigs   117.20 degs A|90:NE=CZ=NH2 (ARG)
 WARNING:        11 planes.       Worst is     7.0 sigs     0.14 Angs A|401:O7=C3=C6=O6 (CIT)
 WARNING:         1 idealD contacts. Worst     5.1 sigs     2.53 Angs A|91:CB=O (ASP)
 WARNING:       107 Bcorrel.      Worst is    18.2 sigs    45.59 Angs^2 B|344:C(LYS)=B|345:N(LEU)

 Weighted rms      0.0136         1.465  (temporary rms bond and angle for BUSTER to pick up!)
 Weighted intra-residual rms(bond) for StdProtein (excluding H)      0.0140
 Weighted intra-residual rms(bond) for Buffer     (excluding H)      0.0273

 ############### Running initial check


 Initial Geometry Sanity Check

 Check for duplicate bonds: no duplicates found
 
 Check for duplicate or missing bond angles: all are OK
 Check for bond between an atom and itself not involving symmetry:  all are OK
   There are no bad contacts where Delta/sigma is greater than     6.5
   Worst contact dist=   2.533 std=   2.753 sigma=   0.043 abs(Delta/sigma)=    5.121 ats A|91:CB (ASP) A|91:O (ASP)
   It is likely that the geometry libraries, sequence info and links match the input molecule
 GEOMETRY CHECK OVERALL:  PASS


 ############### Creating geometry/screen report


 ############### Option "tao" - run Tao optimizer

 TAO: Toolkit for Advanced Optimization. Reference: 
     Munson, T., Sarich, J., Wild, S., Benson, S., McInnes, L.C. (2018)
     "TAO User Manual (Revision 3.9)", Mathematics and Computer Science
     Division, Argonne National Laboratory
     ANL/MCS-TM-322, http://www.mcs.anl.gov/petsc/
 
 Have found -keeppdb flag so will write out a .pdb file every 1000000 its during opt
 Will apply TAO optimization 
 Maximum number of iterations      100
 Convergence test on Grms       4.0000 (-glim)
 Limit on rms displacement to original position (rmsD2init): NONE test inactive (-dlim)

    analysing argument no 0 : -autoncs with j=0
    analysing argument no 1 : -hydrogens_input_occ2zero with j=0
    analysing argument no 2 : -hydrogens_output_occ2heavy with j=0
    analysing argument no 3 : -screen with j=0
    analysing argument no 4 : 100 with j=0
    analysing argument no 5 : -screen_sigma with j=0
    analysing argument no 6 : 3.0 with j=0
    analysing argument no 7 : -keeppdb with j=0
    analysing argument no 8 : 1000000 with j=0
    analysing argument no 9 : -taomonitor_verbose with j=0
    found argument starting with "-tao" : -taomonitor_verbose
    analysing argument no 10 : 2 with j=1
    found value after -tao/-options_/-viewer_ argument : "2"
 PETSC/TAO: setting option "-taomonitor_verbose" = "2"


   MaxLik Scaling Cycle 0003
   Iters     Tot_Funct    Xray_Funct    Geom_Funct          Grms     rmsD2init     maxD2init             R         Rfree           LLG       LLGfree     rms(bond)    rms(angle)       Opttag_H
   Iters             K   B_IMPF_FRAG        K_MISS   B_TEMP_MISS   K_IMPF_MISS   B_IMPF_MISS        K_SOLV   B_TEMP_SOLV   K_IMPF_SOLV   B_IMPF_SOLV        B_TEMP     B_TEMP_11     B_TEMP_12     B_TEMP_22     B_TEMP_13     B_TEMP_23     B_TEMP_33      K_WILSON      B_WILSON      FGCYCLES       Scatag_H
   Iters          BOND         ANGLE      IMPROPER       TORSION    SINTORSION      TRIGONAL        PLANAR  BCORRELATION       CONTACT     IDEALDIST        CHIRAL    SIMILARITY       OCCSSUM  UTILDISTANCE     UTILANGLE   UTILTORSION        RHOFIT        ADP-01        ADP-02        ADP-03        ADP-04        ADP-05        ADP-06        ADP-07        ADP-08        ADP-09        ADP-10      RHOFITPC            QM       Funtag_H
       0  4.607042E+05  1.246057E+05  3.360985E+05       715.856  0.000000E+00  0.000000E+00      0.181625      0.223118  0.000000E+00  0.000000E+00     0.0135555       1.46537       Opttag_D
       0       75.9752       1.28951       1.00000  0.000000E+00       1.00000  0.000000E+00      0.918499       84.3659       1.00000       32.2156  0.000000E+00       9.15000  0.000000E+00       9.15000  0.000000E+00  0.000000E+00      -18.3000       76.6492       2.58297        2       Scatag_D
       0       5161.37       11703.8  0.000000E+00  0.000000E+00       2196.42  0.000000E+00       14444.4  1.762010E+05  0.000000E+00       21899.7       8.06546  1.044838E+05 -1.000000E+10 -1.000000E+10 -1.000000E+10 -1.000000E+10 -1.000000E+10  0.000000E+00  0.000000E+00  0.000000E+00  0.000000E+00  0.000000E+00  0.000000E+00  0.000000E+00  0.000000E+00  0.000000E+00  0.000000E+00  0.000000E+00  0.000000E+00       Funtag_D
   Geometry Restraint Deviations SCREEN Information

 

   MaxLik Scaling Cycle 0004

   MaxLik Scaling Cycle 0005

   Wilson plot

   Statistics in resolution bins after refinement cycle 0001
```


|  |  |
| --- | --- |
|  | - Rxpct factors plot- Rxpct factors table- <Log-Lik Gain>- Ln Sigma A- Correlation Coefficients |


```
   Iters     Tot_Funct    Xray_Funct    Geom_Funct          Grms     rmsD2init     maxD2init             R         Rfree           LLG       LLGfree     rms(bond)    rms(angle)
   Iters             K   B_IMPF_FRAG        K_MISS   B_TEMP_MISS   K_IMPF_MISS   B_IMPF_MISS        K_SOLV   B_TEMP_SOLV   K_IMPF_SOLV   B_IMPF_SOLV        B_TEMP     B_TEMP_11     B_TEMP_12     B_TEMP_22     B_TEMP_13     B_TEMP_23     B_TEMP_33      K_WILSON      B_WILSON      FGCYCLES
   Iters          BOND         ANGLE      IMPROPER       TORSION    SINTORSION      TRIGONAL        PLANAR  BCORRELATION       CONTACT     IDEALDIST        CHIRAL    SIMILARITY       OCCSSUM  UTILDISTANCE     UTILANGLE   UTILTORSION        RHOFIT        ADP-01        ADP-02        ADP-03        ADP-04        ADP-05        ADP-06        ADP-07        ADP-08        ADP-09        ADP-10      RHOFITPC            QM
       1  3.646627E+05  1.253571E+05  2.393056E+05       503.452    0.00726903     0.0763758      0.183015      0.222138   -0.00655649    0.00903140     0.0103562       1.33092       Opttag_D
       1       75.8801       1.34762       1.00000  0.000000E+00       1.00000  0.000000E+00      0.919425       84.0500       1.00000       31.9074  0.000000E+00       9.44192  0.000000E+00       9.44192  0.000000E+00  0.000000E+00      -18.8838       76.6492       2.58297        2       Scatag_D
       1       3012.57       9654.58  0.000000E+00  0.000000E+00       2184.16  0.000000E+00       12408.0       96995.6  0.000000E+00       20154.8       11.0515       94885.0 -1.000000E+10 -1.000000E+10 -1.000000E+10 -1.000000E+10 -1.000000E+10  0.000000E+00  0.000000E+00  0.000000E+00  0.000000E+00  0.000000E+00  0.000000E+00  0.000000E+00  0.000000E+00  0.000000E+00  0.000000E+00  0.000000E+00  0.000000E+00       Funtag_D
   Geometry Restraint Deviations SCREEN Information

 

   MaxLik Scaling Cycle 0006

   Wilson plot

   Statistics in resolution bins after refinement cycle 0002
```


|  |  |
| --- | --- |
|  | - Rxpct factors plot- Rxpct factors table- <Log-Lik Gain>- Ln Sigma A- Correlation Coefficients |


```
   Iters     Tot_Funct    Xray_Funct    Geom_Funct          Grms     rmsD2init     maxD2init             R         Rfree           LLG       LLGfree     rms(bond)    rms(angle)
   Iters             K   B_IMPF_FRAG        K_MISS   B_TEMP_MISS   K_IMPF_MISS   B_IMPF_MISS        K_SOLV   B_TEMP_SOLV   K_IMPF_SOLV   B_IMPF_SOLV        B_TEMP     B_TEMP_11     B_TEMP_12     B_TEMP_22     B_TEMP_13     B_TEMP_23     B_TEMP_33      K_WILSON      B_WILSON      FGCYCLES
   Iters          BOND         ANGLE      IMPROPER       TORSION    SINTORSION      TRIGONAL        PLANAR  BCORRELATION       CONTACT     IDEALDIST        CHIRAL    SIMILARITY       OCCSSUM  UTILDISTANCE     UTILANGLE   UTILTORSION        RHOFIT        ADP-01        ADP-02        ADP-03        ADP-04        ADP-05        ADP-06        ADP-07        ADP-08        ADP-09        ADP-10      RHOFITPC            QM
       2  3.217595E+05  1.257418E+05  1.960177E+05       351.977     0.0127699     0.0545884      0.183831      0.221606   -0.00991318     0.0136188    0.00927861       1.26978       Opttag_D
       2       75.8360       1.37687       1.00000  0.000000E+00       1.00000  0.000000E+00      0.920306       83.4891       1.00000       31.8781  0.000000E+00       9.60085  0.000000E+00       9.60085  0.000000E+00  0.000000E+00      -19.2017       76.6492       2.58297        1       Scatag_D
       2       2418.23       8787.97  0.000000E+00  0.000000E+00       2172.89  0.000000E+00       10890.1       66248.2  0.000000E+00       18932.9       26.8779       86540.5 -1.000000E+10 -1.000000E+10 -1.000000E+10 -1.000000E+10 -1.000000E+10  0.000000E+00  0.000000E+00  0.000000E+00  0.000000E+00  0.000000E+00  0.000000E+00  0.000000E+00  0.000000E+00  0.000000E+00  0.000000E+00  0.000000E+00  0.000000E+00       Funtag_D
   Geometry Restraint Deviations SCREEN Information

 
       3  2.906962E+05  1.263686E+05  1.643276E+05       297.345     0.0219542     0.0766233      0.185180      0.220781    -0.0153818     0.0195900     0.0108309       1.18645       Opttag_D
       3       75.8040       1.42571       1.00000  0.000000E+00       1.00000  0.000000E+00      0.921698       82.5122       1.00000       31.9917  0.000000E+00       9.81244  0.000000E+00       9.81244  0.000000E+00  0.000000E+00      -19.6249       76.6492       2.58297        1       Scatag_D
       3       3295.06       7672.45  0.000000E+00  0.000000E+00       2158.37  0.000000E+00       8858.55       51044.1  0.000000E+00       17379.5       79.6877       73840.0 -1.000000E+10 -1.000000E+10 -1.000000E+10 -1.000000E+10 -1.000000E+10  0.000000E+00  0.000000E+00  0.000000E+00  0.000000E+00  0.000000E+00  0.000000E+00  0.000000E+00  0.000000E+00  0.000000E+00  0.000000E+00  0.000000E+00  0.000000E+00       Funtag_D
       4  2.719193E+05  1.268231E+05  1.450961E+05       269.009     0.0295063     0.0982781      0.186219      0.220000    -0.0193478     0.0240971     0.0118356       1.23557       Opttag_D
       4       75.7663       1.45716       1.00000  0.000000E+00       1.00000  0.000000E+00      0.922502       81.6523       1.00000       32.1987  0.000000E+00       9.94457  0.000000E+00       9.94457  0.000000E+00  0.000000E+00      -19.8891       76.6492       2.58297        1       Scatag_D
       4       3934.73       8320.88  0.000000E+00  0.000000E+00       2149.60  0.000000E+00       7554.79       42629.0  0.000000E+00       16431.9       119.025       63956.2 -1.000000E+10 -1.000000E+10 -1.000000E+10 -1.000000E+10 -1.000000E+10  0.000000E+00  0.000000E+00  0.000000E+00  0.000000E+00  0.000000E+00  0.000000E+00  0.000000E+00  0.000000E+00  0.000000E+00  0.000000E+00  0.000000E+00  0.000000E+00       Funtag_D
       5  2.552570E+05  1.271797E+05  1.280773E+05       225.756     0.0368798      0.124773      0.187104      0.219262    -0.0224592     0.0279659     0.0114967       1.21975       Opttag_D
       5       75.7141       1.47722       1.00000  0.000000E+00       1.00000  0.000000E+00      0.922985       80.7815       1.00000       32.4724  0.000000E+00       10.0381  0.000000E+00       10.0381  0.000000E+00  0.000000E+00      -20.0763       76.6492       2.58297        1       Scatag_D
       5       3712.59       8109.07  0.000000E+00  0.000000E+00       2143.52  0.000000E+00       6600.04       36145.5  0.000000E+00       15744.0       129.696       55492.8 -1.000000E+10 -1.000000E+10 -1.000000E+10 -1.000000E+10 -1.000000E+10  0.000000E+00  0.000000E+00  0.000000E+00  0.000000E+00  0.000000E+00  0.000000E+00  0.000000E+00  0.000000E+00  0.000000E+00  0.000000E+00  0.000000E+00  0.000000E+00       Funtag_D
       6  2.409184E+05  1.279093E+05  1.130091E+05       329.337     0.0510310      0.180322      0.188865      0.218166    -0.0288252     0.0338867     0.0112486       1.44127       Opttag_D
       6       75.6258       1.52118       1.00000  0.000000E+00       1.00000  0.000000E+00      0.923656       79.1836       1.00000       33.0921  0.000000E+00       10.1772  0.000000E+00       10.1772  0.000000E+00  0.000000E+00      -20.3545       76.6492       2.58297        1       Scatag_D
       6       3554.08       11322.0  0.000000E+00  0.000000E+00       2137.42  0.000000E+00       5382.88       31071.0  0.000000E+00       14864.7       132.045       44544.9 -1.000000E+10 -1.000000E+10 -1.000000E+10 -1.000000E+10 -1.000000E+10  0.000000E+00  0.000000E+00  0.000000E+00  0.000000E+00  0.000000E+00  0.000000E+00  0.000000E+00  0.000000E+00  0.000000E+00  0.000000E+00  0.000000E+00  0.000000E+00       Funtag_D
       7  2.298433E+05  1.280455E+05  1.017979E+05       175.062     0.0546604      0.201095      0.189209      0.218019    -0.0300135     0.0350326    0.00987997       1.23572       Opttag_D
       7       75.5798       1.52665       1.00000  0.000000E+00       1.00000  0.000000E+00      0.923608       78.8047       1.00000       33.2196  0.000000E+00       10.2007  0.000000E+00       10.2007  0.000000E+00  0.000000E+00      -20.4014       76.6492       2.58297        1       Scatag_D
       7       2741.85       8322.80  0.000000E+00  0.000000E+00       2136.78  0.000000E+00       5185.82       26761.2  0.000000E+00       14667.3       100.649       41881.5 -1.000000E+10 -1.000000E+10 -1.000000E+10 -1.000000E+10 -1.000000E+10  0.000000E+00  0.000000E+00  0.000000E+00  0.000000E+00  0.000000E+00  0.000000E+00  0.000000E+00  0.000000E+00  0.000000E+00  0.000000E+00  0.000000E+00  0.000000E+00       Funtag_D
       8  2.243845E+05  1.280245E+05       96360.0       145.602     0.0556209      0.209693      0.189189      0.218051    -0.0298301     0.0350841    0.00920743       1.21767       Opttag_D
       8       75.5467       1.52176       1.00000  0.000000E+00       1.00000  0.000000E+00      0.923454       78.7226       1.00000       33.2078  0.000000E+00       10.2013  0.000000E+00       10.2013  0.000000E+00  0.000000E+00      -20.4026       76.6492       2.58297        1       Scatag_D
       8       2381.27       8081.50  0.000000E+00  0.000000E+00       2135.12  0.000000E+00       5158.95       23548.9  0.000000E+00       14594.8       74.3581       40385.2 -1.000000E+10 -1.000000E+10 -1.000000E+10 -1.000000E+10 -1.000000E+10  0.000000E+00  0.000000E+00  0.000000E+00  0.000000E+00  0.000000E+00  0.000000E+00  0.000000E+00  0.000000E+00  0.000000E+00  0.000000E+00  0.000000E+00  0.000000E+00       Funtag_D
       9  2.165507E+05  1.282277E+05       88323.0       169.085     0.0612198      0.251260      0.189728      0.218189    -0.0316036     0.0355613    0.00858927       1.21667       Opttag_D
       9       75.4671       1.52963       1.00000  0.000000E+00       1.00000  0.000000E+00      0.923382       78.1312       1.00000       33.3646  0.000000E+00       10.2364  0.000000E+00       10.2364  0.000000E+00  0.000000E+00      -20.4727       76.6492       2.58297        1       Scatag_D
       9       2072.26       8068.18  0.000000E+00  0.000000E+00       2130.63  0.000000E+00       4890.41       20640.5  0.000000E+00       14282.9       53.9695       36184.1 -1.000000E+10 -1.000000E+10 -1.000000E+10 -1.000000E+10 -1.000000E+10  0.000000E+00  0.000000E+00  0.000000E+00  0.000000E+00  0.000000E+00  0.000000E+00  0.000000E+00  0.000000E+00  0.000000E+00  0.000000E+00  0.000000E+00  0.000000E+00       Funtag_D
      10  2.142419E+05  1.287615E+05       85480.4       303.701     0.0712197      0.323568      0.191043      0.218673    -0.0362608     0.0360933    0.00926755       1.39864       Opttag_D
      10       75.3779       1.56258       1.00000  0.000000E+00       1.00000  0.000000E+00      0.923557       77.0288       1.00000       33.7736  0.000000E+00       10.3030  0.000000E+00       10.3030  0.000000E+00  0.000000E+00      -20.6059       76.6492       2.58297        1       Scatag_D
      10       2412.47       10662.2  0.000000E+00  0.000000E+00       2126.17  0.000000E+00       4445.07       19330.3  0.000000E+00       13844.0       56.8530       32603.4 -1.000000E+10 -1.000000E+10 -1.000000E+10 -1.000000E+10 -1.000000E+10  0.000000E+00  0.000000E+00  0.000000E+00  0.000000E+00  0.000000E+00  0.000000E+00  0.000000E+00  0.000000E+00  0.000000E+00  0.000000E+00  0.000000E+00  0.000000E+00       Funtag_D
      11  2.085423E+05  1.286940E+05       79848.4       113.524     0.0699785      0.313906      0.190895      0.218663    -0.0356718     0.0359449    0.00816217       1.15673       Opttag_D
      11       75.3812       1.55665       1.00000  0.000000E+00       1.00000  0.000000E+00      0.923575       77.0849       1.00000       33.7356  0.000000E+00       10.3040  0.000000E+00       10.3040  0.000000E+00  0.000000E+00      -20.6080       76.6492       2.58297        1       Scatag_D
      11       1871.30       7292.79  0.000000E+00  0.000000E+00       2124.98  0.000000E+00       4435.41       17774.4  0.000000E+00       13847.6       60.5152       32441.3 -1.000000E+10 -1.000000E+10 -1.000000E+10 -1.000000E+10 -1.000000E+10  0.000000E+00  0.000000E+00  0.000000E+00  0.000000E+00  0.000000E+00  0.000000E+00  0.000000E+00  0.000000E+00  0.000000E+00  0.000000E+00  0.000000E+00  0.000000E+00       Funtag_D
      12  2.057698E+05  1.287971E+05       76972.7       88.8301     0.0715552      0.320833      0.191157      0.218808    -0.0365719     0.0359301    0.00796548       1.14208       Opttag_D
      12       75.3622       1.56171       1.00000  0.000000E+00       1.00000  0.000000E+00      0.923669       76.8058       1.00000       33.8320  0.000000E+00       10.3265  0.000000E+00       10.3265  0.000000E+00  0.000000E+00      -20.6531       76.6492       2.58297        1       Scatag_D
      12       1782.20       7109.21  0.000000E+00  0.000000E+00       2122.96  0.000000E+00       4294.99       16240.4  0.000000E+00       13736.2       67.1579       31619.5 -1.000000E+10 -1.000000E+10 -1.000000E+10 -1.000000E+10 -1.000000E+10  0.000000E+00  0.000000E+00  0.000000E+00  0.000000E+00  0.000000E+00  0.000000E+00  0.000000E+00  0.000000E+00  0.000000E+00  0.000000E+00  0.000000E+00  0.000000E+00       Funtag_D
      13  2.023563E+05  1.290767E+05       73279.5       111.804     0.0759406      0.341099      0.191850      0.219164    -0.0390117     0.0358944    0.00796324       1.14956       Opttag_D
      13       75.3139       1.57724       1.00000  0.000000E+00       1.00000  0.000000E+00      0.923812       76.1530       1.00000       34.0697  0.000000E+00       10.3742  0.000000E+00       10.3742  0.000000E+00  0.000000E+00      -20.7484       76.6492       2.58297        1       Scatag_D
      13       1781.20       7202.66  0.000000E+00  0.000000E+00       2119.44  0.000000E+00       4016.27       14573.6  0.000000E+00       13498.9       74.1038       30013.4 -1.000000E+10 -1.000000E+10 -1.000000E+10 -1.000000E+10 -1.000000E+10  0.000000E+00  0.000000E+00  0.000000E+00  0.000000E+00  0.000000E+00  0.000000E+00  0.000000E+00  0.000000E+00  0.000000E+00  0.000000E+00  0.000000E+00  0.000000E+00       Funtag_D
      14  1.989130E+05  1.294477E+05       69465.2       173.867     0.0813666      0.412900      0.192774      0.219716    -0.0422488     0.0357183    0.00822163       1.19247       Opttag_D
      14       75.2382       1.59623       1.00000  0.000000E+00       1.00000  0.000000E+00      0.923908       75.2575       1.00000       34.3855  0.000000E+00       10.4426  0.000000E+00       10.4426  0.000000E+00  0.000000E+00      -20.8852       76.6492       2.58297        1       Scatag_D
      14       1898.67       7750.46  0.000000E+00  0.000000E+00       2113.96  0.000000E+00       3657.08       12898.4  0.000000E+00       13183.3       72.2099       27891.1 -1.000000E+10 -1.000000E+10 -1.000000E+10 -1.000000E+10 -1.000000E+10  0.000000E+00  0.000000E+00  0.000000E+00  0.000000E+00  0.000000E+00  0.000000E+00  0.000000E+00  0.000000E+00  0.000000E+00  0.000000E+00  0.000000E+00  0.000000E+00       Funtag_D
      15  1.959189E+05  1.298385E+05       66080.4       115.280     0.0869505      0.491475      0.193739      0.220312    -0.0456584     0.0351661    0.00812437       1.11874       Opttag_D
      15       75.1678       1.61796       1.00000  0.000000E+00       1.00000  0.000000E+00      0.923944       74.3978       1.00000       34.7202  0.000000E+00       10.5010  0.000000E+00       10.5010  0.000000E+00  0.000000E+00      -21.0020       76.6492       2.58297        1       Scatag_D
      15       1854.01       6821.67  0.000000E+00  0.000000E+00       2111.16  0.000000E+00       3390.82       12157.9  0.000000E+00       12918.9       65.1388       26760.8 -1.000000E+10 -1.000000E+10 -1.000000E+10 -1.000000E+10 -1.000000E+10  0.000000E+00  0.000000E+00  0.000000E+00  0.000000E+00  0.000000E+00  0.000000E+00  0.000000E+00  0.000000E+00  0.000000E+00  0.000000E+00  0.000000E+00  0.000000E+00       Funtag_D
      16  1.938427E+05  1.297858E+05       64056.9       74.3521     0.0863720      0.489739      0.193627      0.220321    -0.0451987     0.0351053    0.00774688       1.08804       Opttag_D
      16       75.1502       1.61119       1.00000  0.000000E+00       1.00000  0.000000E+00      0.923823       74.3953       1.00000       34.6908  0.000000E+00       10.5025  0.000000E+00       10.5025  0.000000E+00  0.000000E+00      -21.0050       76.6492       2.58297        1       Scatag_D
      16       1685.72       6452.43  0.000000E+00  0.000000E+00       2109.92  0.000000E+00       3376.30       11349.6  0.000000E+00       12877.1       57.0159       26148.9 -1.000000E+10 -1.000000E+10 -1.000000E+10 -1.000000E+10 -1.000000E+10  0.000000E+00  0.000000E+00  0.000000E+00  0.000000E+00  0.000000E+00  0.000000E+00  0.000000E+00  0.000000E+00  0.000000E+00  0.000000E+00  0.000000E+00  0.000000E+00       Funtag_D
      17  1.910009E+05  1.297769E+05       61224.1       85.3987     0.0867229      0.498434      0.193665      0.220531    -0.0451205     0.0346213    0.00736800       1.07735       Opttag_D
      17       75.0938       1.60222       1.00000  0.000000E+00       1.00000  0.000000E+00      0.923568       74.1410       1.00000       34.7308  0.000000E+00       10.5193  0.000000E+00       10.5193  0.000000E+00  0.000000E+00      -21.0387       76.6492       2.58297        1       Scatag_D
      17       1524.86       6326.25  0.000000E+00  0.000000E+00       2106.65  0.000000E+00       3304.41       10341.5  0.000000E+00       12724.9       44.5088       24851.0 -1.000000E+10 -1.000000E+10 -1.000000E+10 -1.000000E+10 -1.000000E+10  0.000000E+00  0.000000E+00  0.000000E+00  0.000000E+00  0.000000E+00  0.000000E+00  0.000000E+00  0.000000E+00  0.000000E+00  0.000000E+00  0.000000E+00  0.000000E+00       Funtag_D
      18  1.896994E+05  1.299614E+05       59738.0       149.799     0.0897754      0.520704      0.194191      0.221018    -0.0467308     0.0336984    0.00746411       1.11654       Opttag_D
      18       75.0273       1.60669       1.00000  0.000000E+00       1.00000  0.000000E+00      0.923433       73.5241       1.00000       34.9525  0.000000E+00       10.5546  0.000000E+00       10.5546  0.000000E+00  0.000000E+00      -21.1092       76.6492       2.58297        1       Scatag_D
      18       1564.91       6794.86  0.000000E+00  0.000000E+00       2104.13  0.000000E+00       3186.66       9545.13  0.000000E+00       12501.5       42.6718       23998.1 -1.000000E+10 -1.000000E+10 -1.000000E+10 -1.000000E+10 -1.000000E+10  0.000000E+00  0.000000E+00  0.000000E+00  0.000000E+00  0.000000E+00  0.000000E+00  0.000000E+00  0.000000E+00  0.000000E+00  0.000000E+00  0.000000E+00  0.000000E+00       Funtag_D
      19  1.879282E+05  1.300281E+05       57900.1       69.9654     0.0908659      0.520410      0.194389      0.221204    -0.0473127     0.0333376    0.00716413       1.04986       Opttag_D
      19       75.0082       1.60855       1.00000  0.000000E+00       1.00000  0.000000E+00      0.923427       73.2933       1.00000       35.0404  0.000000E+00       10.5665  0.000000E+00       10.5665  0.000000E+00  0.000000E+00      -21.1329       76.6492       2.58297        1       Scatag_D
      19       1441.65       6007.47  0.000000E+00  0.000000E+00       2103.11  0.000000E+00       3146.01       9037.47  0.000000E+00       12414.6       45.4035       23704.4 -1.000000E+10 -1.000000E+10 -1.000000E+10 -1.000000E+10 -1.000000E+10  0.000000E+00  0.000000E+00  0.000000E+00  0.000000E+00  0.000000E+00  0.000000E+00  0.000000E+00  0.000000E+00  0.000000E+00  0.000000E+00  0.000000E+00  0.000000E+00       Funtag_D
      20  1.863874E+05  1.301648E+05       56222.6       58.9939     0.0929124      0.513834      0.194780      0.221516    -0.0485054     0.0327986    0.00698277       1.03763       Opttag_D
      20       74.9813       1.61403       1.00000  0.000000E+00       1.00000  0.000000E+00      0.923478       72.8705       1.00000       35.2115  0.000000E+00       10.5887  0.000000E+00       10.5887  0.000000E+00  0.000000E+00      -21.1774       76.6492       2.58297        1       Scatag_D
      20       1369.58       5868.42  0.000000E+00  0.000000E+00       2101.45  0.000000E+00       3067.15       8306.40  0.000000E+00       12258.5       54.1431       23197.0 -1.000000E+10 -1.000000E+10 -1.000000E+10 -1.000000E+10 -1.000000E+10  0.000000E+00  0.000000E+00  0.000000E+00  0.000000E+00  0.000000E+00  0.000000E+00  0.000000E+00  0.000000E+00  0.000000E+00  0.000000E+00  0.000000E+00  0.000000E+00       Funtag_D
      21  1.852199E+05  1.303306E+05       54889.3       66.6578     0.0951421      0.503598      0.195235      0.221831    -0.0499520     0.0323981    0.00690013       1.03237       Opttag_D
      21       74.9553       1.62203       1.00000  0.000000E+00       1.00000  0.000000E+00      0.923541       72.4222       1.00000       35.3980  0.000000E+00       10.6148  0.000000E+00       10.6148  0.000000E+00  0.000000E+00      -21.2296       76.6492       2.58297        1       Scatag_D
      21       1337.35       5808.98  0.000000E+00  0.000000E+00       2099.82  0.000000E+00       2984.62       7797.06  0.000000E+00       12096.4       60.5357       22704.5 -1.000000E+10 -1.000000E+10 -1.000000E+10 -1.000000E+10 -1.000000E+10  0.000000E+00  0.000000E+00  0.000000E+00  0.000000E+00  0.000000E+00  0.000000E+00  0.000000E+00  0.000000E+00  0.000000E+00  0.000000E+00  0.000000E+00  0.000000E+00       Funtag_D
      22  1.841681E+05  1.307662E+05       53401.9       173.156      0.100740      0.590963      0.196418      0.222598    -0.0537533     0.0317145    0.00720668       1.11165       Opttag_D
      22       74.8982       1.64444       1.00000  0.000000E+00       1.00000  0.000000E+00      0.923705       71.3217       1.00000       35.8812  0.000000E+00       10.6803  0.000000E+00       10.6803  0.000000E+00  0.000000E+00      -21.3606       76.6492       2.58297        1       Scatag_D
      22       1458.82       6735.42  0.000000E+00  0.000000E+00       2096.61  0.000000E+00       2801.23       6874.66  0.000000E+00       11717.9       63.6482       21653.6 -1.000000E+10 -1.000000E+10 -1.000000E+10 -1.000000E+10 -1.000000E+10  0.000000E+00  0.000000E+00  0.000000E+00  0.000000E+00  0.000000E+00  0.000000E+00  0.000000E+00  0.000000E+00  0.000000E+00  0.000000E+00  0.000000E+00  0.000000E+00       Funtag_D
      23  1.822219E+05  1.309009E+05       51321.0       57.0220      0.102413      0.633009      0.196779      0.222814    -0.0549281     0.0315963    0.00685825       1.00646       Opttag_D
      23       74.8816       1.65162       1.00000  0.000000E+00       1.00000  0.000000E+00      0.923747       70.9961       1.00000       36.0295  0.000000E+00       10.6999  0.000000E+00       10.6999  0.000000E+00  0.000000E+00      -21.3998       76.6492       2.58297        1       Scatag_D
      23       1321.17       5521.08  0.000000E+00  0.000000E+00       2095.89  0.000000E+00       2751.29       6594.30  0.000000E+00       11605.7       61.0663       21370.5 -1.000000E+10 -1.000000E+10 -1.000000E+10 -1.000000E+10 -1.000000E+10  0.000000E+00  0.000000E+00  0.000000E+00  0.000000E+00  0.000000E+00  0.000000E+00  0.000000E+00  0.000000E+00  0.000000E+00  0.000000E+00  0.000000E+00  0.000000E+00       Funtag_D
      24  1.813872E+05  1.309148E+05       50472.4       44.7871      0.102605      0.650243      0.196828      0.222793    -0.0550493     0.0318169    0.00671342       1.00074       Opttag_D
      24       74.8725       1.65099       1.00000  0.000000E+00       1.00000  0.000000E+00      0.923730       70.9246       1.00000       36.0507  0.000000E+00       10.7069  0.000000E+00       10.7069  0.000000E+00  0.000000E+00      -21.4138       76.6492       2.58297        1       Scatag_D
      24       1265.96       5458.46  0.000000E+00  0.000000E+00       2095.05  0.000000E+00       2725.54       6268.45  0.000000E+00       11549.4       52.7911       21056.8 -1.000000E+10 -1.000000E+10 -1.000000E+10 -1.000000E+10 -1.000000E+10  0.000000E+00  0.000000E+00  0.000000E+00  0.000000E+00  0.000000E+00  0.000000E+00  0.000000E+00  0.000000E+00  0.000000E+00  0.000000E+00  0.000000E+00  0.000000E+00       Funtag_D
      25  1.803314E+05  1.310225E+05       49309.0       54.6520      0.104162      0.706284      0.197143      0.222914    -0.0559889     0.0319344    0.00658578      0.998310       Opttag_D
      25       74.8453       1.65406       1.00000  0.000000E+00       1.00000  0.000000E+00      0.923723       70.5870       1.00000       36.1871  0.000000E+00       10.7292  0.000000E+00       10.7292  0.000000E+00  0.000000E+00      -21.4583       76.6492       2.58297        1       Scatag_D
      25       1218.28       5432.03  0.000000E+00  0.000000E+00       2093.60  0.000000E+00       2663.19       5916.32  0.000000E+00       11389.7       42.8299       20553.0 -1.000000E+10 -1.000000E+10 -1.000000E+10 -1.000000E+10 -1.000000E+10  0.000000E+00  0.000000E+00  0.000000E+00  0.000000E+00  0.000000E+00  0.000000E+00  0.000000E+00  0.000000E+00  0.000000E+00  0.000000E+00  0.000000E+00  0.000000E+00       Funtag_D
      26  1.797978E+05  1.311730E+05       48624.8       130.770      0.106749      0.776252      0.197609      0.223132    -0.0573022     0.0317212    0.00664493       1.04716       Opttag_D
      26       74.8027       1.65730       1.00000  0.000000E+00       1.00000  0.000000E+00      0.923730       70.0589       1.00000       36.3965  0.000000E+00       10.7582  0.000000E+00       10.7582  0.000000E+00  0.000000E+00      -21.5164       76.6492       2.58297        1       Scatag_D
      26       1240.26       5976.61  0.000000E+00  0.000000E+00       2092.35  0.000000E+00       2585.65       5453.16  0.000000E+00       11156.2       41.8900       20078.7 -1.000000E+10 -1.000000E+10 -1.000000E+10 -1.000000E+10 -1.000000E+10  0.000000E+00  0.000000E+00  0.000000E+00  0.000000E+00  0.000000E+00  0.000000E+00  0.000000E+00  0.000000E+00  0.000000E+00  0.000000E+00  0.000000E+00  0.000000E+00       Funtag_D
      27  1.787558E+05  1.312213E+05       47534.6       48.3023      0.107582      0.792457      0.197758      0.223224    -0.0577236     0.0315742    0.00641185      0.985594       Opttag_D
      27       74.7909       1.65868       1.00000  0.000000E+00       1.00000  0.000000E+00      0.923743       69.8991       1.00000       36.4608  0.000000E+00       10.7665  0.000000E+00       10.7665  0.000000E+00  0.000000E+00      -21.5331       76.6492       2.58297        1       Scatag_D
      27       1154.78       5294.54  0.000000E+00  0.000000E+00       2091.96  0.000000E+00       2565.10       5319.04  0.000000E+00       11088.2       42.8614       19978.1 -1.000000E+10 -1.000000E+10 -1.000000E+10 -1.000000E+10 -1.000000E+10  0.000000E+00  0.000000E+00  0.000000E+00  0.000000E+00  0.000000E+00  0.000000E+00  0.000000E+00  0.000000E+00  0.000000E+00  0.000000E+00  0.000000E+00  0.000000E+00       Funtag_D
      28  1.781861E+05  1.312631E+05       46923.0       34.4731      0.108468      0.800684      0.197899      0.223329    -0.0580890     0.0313128    0.00631161      0.977412       Opttag_D
      28       74.7774       1.65916       1.00000  0.000000E+00       1.00000  0.000000E+00      0.923770       69.7250       1.00000       36.5236  0.000000E+00       10.7746  0.000000E+00       10.7746  0.000000E+00  0.000000E+00      -21.5492       76.6492       2.58297        1       Scatag_D
      28       1118.95       5206.99  0.000000E+00  0.000000E+00       2091.54  0.000000E+00       2540.20       5108.61  0.000000E+00       11010.1       47.6391       19799.0 -1.000000E+10 -1.000000E+10 -1.000000E+10 -1.000000E+10 -1.000000E+10  0.000000E+00  0.000000E+00  0.000000E+00  0.000000E+00  0.000000E+00  0.000000E+00  0.000000E+00  0.000000E+00  0.000000E+00  0.000000E+00  0.000000E+00  0.000000E+00       Funtag_D
      29  1.776069E+05  1.313372E+05       46269.7       46.6809      0.109973      0.811280      0.198142      0.223501    -0.0587349     0.0309087    0.00624444      0.974634       Opttag_D
      29       74.7568       1.66056       1.00000  0.000000E+00       1.00000  0.000000E+00      0.923819       69.4386       1.00000       36.6296  0.000000E+00       10.7883  0.000000E+00       10.7883  0.000000E+00  0.000000E+00      -21.5766       76.6492       2.58297        1       Scatag_D
      29       1095.26       5177.44  0.000000E+00  0.000000E+00       2090.91  0.000000E+00       2504.04       4900.14  0.000000E+00       10883.5       53.2762       19565.2 -1.000000E+10 -1.000000E+10 -1.000000E+10 -1.000000E+10 -1.000000E+10  0.000000E+00  0.000000E+00  0.000000E+00  0.000000E+00  0.000000E+00  0.000000E+00  0.000000E+00  0.000000E+00  0.000000E+00  0.000000E+00  0.000000E+00  0.000000E+00       Funtag_D
      30  1.768244E+05  1.314709E+05       45353.5       82.4979      0.112717      0.807747      0.198560      0.223838    -0.0599018     0.0301572    0.00622183      0.981494       Opttag_D
      30       74.7253       1.66381       1.00000  0.000000E+00       1.00000  0.000000E+00      0.923923       68.9459       1.00000       36.8178  0.000000E+00       10.8124  0.000000E+00       10.8124  0.000000E+00  0.000000E+00      -21.6247       76.6492       2.58297        1       Scatag_D
      30       1087.35       5250.58  0.000000E+00  0.000000E+00       2089.91  0.000000E+00       2450.02       4529.99  0.000000E+00       10666.3       52.3288       19227.0 -1.000000E+10 -1.000000E+10 -1.000000E+10 -1.000000E+10 -1.000000E+10  0.000000E+00  0.000000E+00  0.000000E+00  0.000000E+00  0.000000E+00  0.000000E+00  0.000000E+00  0.000000E+00  0.000000E+00  0.000000E+00  0.000000E+00  0.000000E+00       Funtag_D
      31  1.761060E+05  1.316414E+05       44464.6       51.5140      0.116004      0.801948      0.199075      0.224242    -0.0613892     0.0292545    0.00619602      0.953478       Opttag_D
      31       74.6965       1.67006       1.00000  0.000000E+00       1.00000  0.000000E+00      0.924040       68.4059       1.00000       37.0476  0.000000E+00       10.8368  0.000000E+00       10.8368  0.000000E+00  0.000000E+00      -21.6735       76.6492       2.58297        1       Scatag_D
      31       1078.34       4955.11  0.000000E+00  0.000000E+00       2089.71  0.000000E+00       2405.97       4301.69  0.000000E+00       10459.1       49.3526       19125.3 -1.000000E+10 -1.000000E+10 -1.000000E+10 -1.000000E+10 -1.000000E+10  0.000000E+00  0.000000E+00  0.000000E+00  0.000000E+00  0.000000E+00  0.000000E+00  0.000000E+00  0.000000E+00  0.000000E+00  0.000000E+00  0.000000E+00  0.000000E+00       Funtag_D
      32  1.756183E+05  1.316650E+05       43953.4       31.6799      0.116630      0.784586      0.199153      0.224312    -0.0615950     0.0291041    0.00611696      0.943627       Opttag_D
      32       74.6908       1.67016       1.00000  0.000000E+00       1.00000  0.000000E+00      0.924069       68.3010       1.00000       37.0884  0.000000E+00       10.8423  0.000000E+00       10.8423  0.000000E+00  0.000000E+00      -21.6845       76.6492       2.58297        1       Scatag_D
      32       1051.00       4853.25  0.000000E+00  0.000000E+00       2089.42  0.000000E+00       2388.67       4140.57  0.000000E+00       10408.5       44.2005       18977.8 -1.000000E+10 -1.000000E+10 -1.000000E+10 -1.000000E+10 -1.000000E+10  0.000000E+00  0.000000E+00  0.000000E+00  0.000000E+00  0.000000E+00  0.000000E+00  0.000000E+00  0.000000E+00  0.000000E+00  0.000000E+00  0.000000E+00  0.000000E+00       Funtag_D
      33  1.750024E+05  1.317192E+05       43283.2       42.8740      0.118058      0.744819      0.199322      0.224461    -0.0620679     0.0288034    0.00604859      0.941095       Opttag_D
      33       74.6785       1.67072       1.00000  0.000000E+00       1.00000  0.000000E+00      0.924137       68.0744       1.00000       37.1784  0.000000E+00       10.8539  0.000000E+00       10.8539  0.000000E+00  0.000000E+00      -21.7079       76.6492       2.58297        1       Scatag_D
      33       1027.64       4827.24  0.000000E+00  0.000000E+00       2088.69  0.000000E+00       2349.51       3976.17  0.000000E+00       10300.4       35.8192       18677.8 -1.000000E+10 -1.000000E+10 -1.000000E+10 -1.000000E+10 -1.000000E+10  0.000000E+00  0.000000E+00  0.000000E+00  0.000000E+00  0.000000E+00  0.000000E+00  0.000000E+00  0.000000E+00  0.000000E+00  0.000000E+00  0.000000E+00  0.000000E+00       Funtag_D
      34  1.746023E+05  1.318258E+05       42776.5       53.2110      0.120367      0.711406      0.199640      0.224718    -0.0629981     0.0282711    0.00604271      0.939244       Opttag_D
      34       74.6638       1.67458       1.00000  0.000000E+00       1.00000  0.000000E+00      0.924259       67.7335       1.00000       37.3242  0.000000E+00       10.8701  0.000000E+00       10.8701  0.000000E+00  0.000000E+00      -21.7402       76.6492       2.58297        1       Scatag_D
      34       1025.64       4808.27  0.000000E+00  0.000000E+00       2088.88  0.000000E+00       2302.97       3879.83  0.000000E+00       10167.5       32.9940       18470.4 -1.000000E+10 -1.000000E+10 -1.000000E+10 -1.000000E+10 -1.000000E+10  0.000000E+00  0.000000E+00  0.000000E+00  0.000000E+00  0.000000E+00  0.000000E+00  0.000000E+00  0.000000E+00  0.000000E+00  0.000000E+00  0.000000E+00  0.000000E+00       Funtag_D
      35  1.741452E+05  1.318934E+05       42251.8       34.8675      0.121852      0.696652      0.199839      0.224867    -0.0635886     0.0279579    0.00594189      0.929512       Opttag_D
      35       74.6554       1.67723       1.00000  0.000000E+00       1.00000  0.000000E+00      0.924352       67.5211       1.00000       37.4143  0.000000E+00       10.8798  0.000000E+00       10.8798  0.000000E+00  0.000000E+00      -21.7597       76.6492       2.58297        1       Scatag_D
      35       991.704       4709.14  0.000000E+00  0.000000E+00       2088.79  0.000000E+00       2270.26       3731.95  0.000000E+00       10084.3       34.8825       18340.7 -1.000000E+10 -1.000000E+10 -1.000000E+10 -1.000000E+10 -1.000000E+10  0.000000E+00  0.000000E+00  0.000000E+00  0.000000E+00  0.000000E+00  0.000000E+00  0.000000E+00  0.000000E+00  0.000000E+00  0.000000E+00  0.000000E+00  0.000000E+00       Funtag_D
      36  1.735393E+05  1.320380E+05       41501.3       35.5641      0.124956      0.681263      0.200262      0.225152    -0.0648496     0.0272683    0.00583783      0.926171       Opttag_D
      36       74.6423       1.68371       1.00000  0.000000E+00       1.00000  0.000000E+00      0.924560       67.0930       1.00000       37.6022  0.000000E+00       10.8982  0.000000E+00       10.8982  0.000000E+00  0.000000E+00      -21.7963       76.6492       2.58297        1       Scatag_D
      36       957.272       4675.36  0.000000E+00  0.000000E+00       2089.40  0.000000E+00       2207.53       3467.50  0.000000E+00       9925.35       42.9608       18136.0 -1.000000E+10 -1.000000E+10 -1.000000E+10 -1.000000E+10 -1.000000E+10  0.000000E+00  0.000000E+00  0.000000E+00  0.000000E+00  0.000000E+00  0.000000E+00  0.000000E+00  0.000000E+00  0.000000E+00  0.000000E+00  0.000000E+00  0.000000E+00       Funtag_D
      37  1.732081E+05  1.321457E+05       41062.4       43.8247      0.127346      0.681980      0.200574      0.225399    -0.0657899     0.0266793    0.00581263      0.926807       Opttag_D
      37       74.6378       1.68909       1.00000  0.000000E+00       1.00000  0.000000E+00      0.924732       66.7821       1.00000       37.7436  0.000000E+00       10.9110  0.000000E+00       10.9110  0.000000E+00  0.000000E+00      -21.8221       76.6492       2.58297        1       Scatag_D
      37       949.024       4681.78  0.000000E+00  0.000000E+00       2089.44  0.000000E+00       2166.81       3339.96  0.000000E+00       9810.97       45.3488       17979.1 -1.000000E+10 -1.000000E+10 -1.000000E+10 -1.000000E+10 -1.000000E+10  0.000000E+00  0.000000E+00  0.000000E+00  0.000000E+00  0.000000E+00  0.000000E+00  0.000000E+00  0.000000E+00  0.000000E+00  0.000000E+00  0.000000E+00  0.000000E+00       Funtag_D
      38  1.728482E+05  1.322232E+05       40625.0       29.7637      0.129134      0.689324      0.200796      0.225598    -0.0664663     0.0261955    0.00574227      0.918098       Opttag_D
      38       74.6372       1.69312       1.00000  0.000000E+00       1.00000  0.000000E+00      0.924864       66.5579       1.00000       37.8480  0.000000E+00       10.9202  0.000000E+00       10.9202  0.000000E+00  0.000000E+00      -21.8403       76.6492       2.58297        1       Scatag_D
      38       926.189       4594.20  0.000000E+00  0.000000E+00       2089.81  0.000000E+00       2139.98       3261.43  0.000000E+00       9726.00       44.3493       17843.0 -1.000000E+10 -1.000000E+10 -1.000000E+10 -1.000000E+10 -1.000000E+10  0.000000E+00  0.000000E+00  0.000000E+00  0.000000E+00  0.000000E+00  0.000000E+00  0.000000E+00  0.000000E+00  0.000000E+00  0.000000E+00  0.000000E+00  0.000000E+00       Funtag_D
      39  1.723840E+05  1.323479E+05       40036.1       32.5068      0.132089      0.711724      0.201152      0.225957    -0.0675539     0.0253042    0.00571628      0.914222       Opttag_D
      39       74.6412       1.69998       1.00000  0.000000E+00       1.00000  0.000000E+00      0.925087       66.2022       1.00000       38.0183  0.000000E+00       10.9345  0.000000E+00       10.9345  0.000000E+00  0.000000E+00      -21.8690       76.6492       2.58297        1       Scatag_D
      39       917.824       4555.49  0.000000E+00  0.000000E+00       2090.28  0.000000E+00       2103.05       3134.53  0.000000E+00       9590.88       38.5805       17605.5 -1.000000E+10 -1.000000E+10 -1.000000E+10 -1.000000E+10 -1.000000E+10  0.000000E+00  0.000000E+00  0.000000E+00  0.000000E+00  0.000000E+00  0.000000E+00  0.000000E+00  0.000000E+00  0.000000E+00  0.000000E+00  0.000000E+00  0.000000E+00       Funtag_D
      40  1.720367E+05  1.324602E+05       39576.5       38.8892      0.134709      0.734765      0.201470      0.226299    -0.0685339     0.0244438    0.00573136      0.913945       Opttag_D
      40       74.6479       1.70655       1.00000  0.000000E+00       1.00000  0.000000E+00      0.925291       65.8946       1.00000       38.1685  0.000000E+00       10.9470  0.000000E+00       10.9470  0.000000E+00  0.000000E+00      -21.8941       76.6492       2.58297        1       Scatag_D
      40       922.672       4552.73  0.000000E+00  0.000000E+00       2091.04  0.000000E+00       2072.34       3009.77  0.000000E+00       9479.93       34.4178       17413.6 -1.000000E+10 -1.000000E+10 -1.000000E+10 -1.000000E+10 -1.000000E+10  0.000000E+00  0.000000E+00  0.000000E+00  0.000000E+00  0.000000E+00  0.000000E+00  0.000000E+00  0.000000E+00  0.000000E+00  0.000000E+00  0.000000E+00  0.000000E+00       Funtag_D
      41  1.716626E+05  1.325627E+05       39099.9       30.8971      0.137020      0.754403      0.201755      0.226617    -0.0694278     0.0236044    0.00568537      0.909048       Opttag_D
      41       74.6557       1.71282       1.00000  0.000000E+00       1.00000  0.000000E+00      0.925481       65.6242       1.00000       38.3015  0.000000E+00       10.9576  0.000000E+00       10.9576  0.000000E+00  0.000000E+00      -21.9152       76.6492       2.58297        1       Scatag_D
      41       907.925       4504.07  0.000000E+00  0.000000E+00       2092.10  0.000000E+00       2042.27       2890.32  0.000000E+00       9385.13       32.7031       17245.4 -1.000000E+10 -1.000000E+10 -1.000000E+10 -1.000000E+10 -1.000000E+10  0.000000E+00  0.000000E+00  0.000000E+00  0.000000E+00  0.000000E+00  0.000000E+00  0.000000E+00  0.000000E+00  0.000000E+00  0.000000E+00  0.000000E+00  0.000000E+00       Funtag_D
      42  1.712569E+05  1.327349E+05       38522.0       42.6499      0.140799      0.781379      0.202225      0.227163    -0.0709309     0.0221507    0.00563046      0.909078       Opttag_D
      42       74.6716       1.72373       1.00000  0.000000E+00       1.00000  0.000000E+00      0.925815       65.1785       1.00000       38.5220  0.000000E+00       10.9737  0.000000E+00       10.9737  0.000000E+00  0.000000E+00      -21.9475       76.6492       2.58297        1       Scatag_D
      42       890.471       4504.37  0.000000E+00  0.000000E+00       2094.15  0.000000E+00       1988.73       2776.79  0.000000E+00       9240.21       34.3693       16992.9 -1.000000E+10 -1.000000E+10 -1.000000E+10 -1.000000E+10 -1.000000E+10  0.000000E+00  0.000000E+00  0.000000E+00  0.000000E+00  0.000000E+00  0.000000E+00  0.000000E+00  0.000000E+00  0.000000E+00  0.000000E+00  0.000000E+00  0.000000E+00       Funtag_D
      43  1.709157E+05  1.327903E+05       38125.4       31.2339      0.142098      0.783979      0.202378      0.227377    -0.0714137     0.0215892    0.00553454      0.902517       Opttag_D
      43       74.6786       1.72688       1.00000  0.000000E+00       1.00000  0.000000E+00      0.925956       65.0123       1.00000       38.5985  0.000000E+00       10.9788  0.000000E+00       10.9788  0.000000E+00  0.000000E+00      -21.9576       76.6492       2.58297        1       Scatag_D
      43       860.389       4439.59  0.000000E+00  0.000000E+00       2094.98  0.000000E+00       1964.53       2704.50  0.000000E+00       9181.82       36.9643       16842.6 -1.000000E+10 -1.000000E+10 -1.000000E+10 -1.000000E+10 -1.000000E+10  0.000000E+00  0.000000E+00  0.000000E+00  0.000000E+00  0.000000E+00  0.000000E+00  0.000000E+00  0.000000E+00  0.000000E+00  0.000000E+00  0.000000E+00  0.000000E+00       Funtag_D
      44  1.706249E+05  1.328037E+05       37821.2       29.0674      0.142593      0.778007      0.202416      0.227468    -0.0715310     0.0213417    0.00547143      0.898953       Opttag_D
      44       74.6824       1.72698       1.00000  0.000000E+00       1.00000  0.000000E+00      0.926038       64.9321       1.00000       38.6277  0.000000E+00       10.9805  0.000000E+00       10.9805  0.000000E+00  0.000000E+00      -21.9611       76.6492       2.58297        1       Scatag_D
      44       840.880       4404.59  0.000000E+00  0.000000E+00       2095.18  0.000000E+00       1951.70       2640.17  0.000000E+00       9148.44       39.4039       16700.9 -1.000000E+10 -1.000000E+10 -1.000000E+10 -1.000000E+10 -1.000000E+10  0.000000E+00  0.000000E+00  0.000000E+00  0.000000E+00  0.000000E+00  0.000000E+00  0.000000E+00  0.000000E+00  0.000000E+00  0.000000E+00  0.000000E+00  0.000000E+00       Funtag_D
      45  1.702742E+05  1.328895E+05       37384.6       46.3334      0.145110      0.837857      0.202652      0.227877    -0.0722798     0.0203159    0.00548637      0.896628       Opttag_D
      45       74.7012       1.73099       1.00000  0.000000E+00       1.00000  0.000000E+00      0.926339       64.6046       1.00000       38.7800  0.000000E+00       10.9878  0.000000E+00       10.9878  0.000000E+00  0.000000E+00      -21.9756       76.6492       2.58297        1       Scatag_D
      45       845.477       4381.84  0.000000E+00  0.000000E+00       2095.59  0.000000E+00       1929.42       2567.61  0.000000E+00       9038.27       39.6112       16486.8 -1.000000E+10 -1.000000E+10 -1.000000E+10 -1.000000E+10 -1.000000E+10  0.000000E+00  0.000000E+00  0.000000E+00  0.000000E+00  0.000000E+00  0.000000E+00  0.000000E+00  0.000000E+00  0.000000E+00  0.000000E+00  0.000000E+00  0.000000E+00       Funtag_D
      46  1.700281E+05  1.329428E+05       37085.3       35.8604      0.146684      0.872984      0.202799      0.228121    -0.0727447     0.0197466    0.00546100      0.887736       Opttag_D
      46       74.7143       1.73379       1.00000  0.000000E+00       1.00000  0.000000E+00      0.926511       64.4137       1.00000       38.8771  0.000000E+00       10.9925  0.000000E+00       10.9925  0.000000E+00  0.000000E+00      -21.9849       76.6492       2.58297        1       Scatag_D
      46       837.678       4295.36  0.000000E+00  0.000000E+00       2095.72  0.000000E+00       1917.46       2497.13  0.000000E+00       8975.79       36.5794       16429.6 -1.000000E+10 -1.000000E+10 -1.000000E+10 -1.000000E+10 -1.000000E+10  0.000000E+00  0.000000E+00  0.000000E+00  0.000000E+00  0.000000E+00  0.000000E+00  0.000000E+00  0.000000E+00  0.000000E+00  0.000000E+00  0.000000E+00  0.000000E+00       Funtag_D
      47  1.698578E+05  1.329125E+05       36945.3       20.3237      0.146092      0.852701      0.202721      0.228016    -0.0724803     0.0200239    0.00539342      0.882254       Opttag_D
      47       74.7116       1.73170       1.00000  0.000000E+00       1.00000  0.000000E+00      0.926454       64.4827       1.00000       38.8405  0.000000E+00       10.9910  0.000000E+00       10.9910  0.000000E+00  0.000000E+00      -21.9820       76.6492       2.58297        1       Scatag_D
      47       817.074       4242.47  0.000000E+00  0.000000E+00       2095.03  0.000000E+00       1917.92       2471.02  0.000000E+00       8990.25       34.5309       16377.0 -1.000000E+10 -1.000000E+10 -1.000000E+10 -1.000000E+10 -1.000000E+10  0.000000E+00  0.000000E+00  0.000000E+00  0.000000E+00  0.000000E+00  0.000000E+00  0.000000E+00  0.000000E+00  0.000000E+00  0.000000E+00  0.000000E+00  0.000000E+00       Funtag_D
      48  1.696667E+05  1.329178E+05       36748.9       15.0218      0.146506      0.850884      0.202736      0.228066    -0.0725266     0.0199491    0.00537988      0.878228       Opttag_D
      48       74.7183       1.73167       1.00000  0.000000E+00       1.00000  0.000000E+00      0.926520       64.4284       1.00000       38.8608  0.000000E+00       10.9933  0.000000E+00       10.9933  0.000000E+00  0.000000E+00      -21.9866       76.6492       2.58297        1       Scatag_D
      48       812.974       4203.84  0.000000E+00  0.000000E+00       2094.10  0.000000E+00       1907.19       2437.51  0.000000E+00       8962.41       33.4772       16297.4 -1.000000E+10 -1.000000E+10 -1.000000E+10 -1.000000E+10 -1.000000E+10  0.000000E+00  0.000000E+00  0.000000E+00  0.000000E+00  0.000000E+00  0.000000E+00  0.000000E+00  0.000000E+00  0.000000E+00  0.000000E+00  0.000000E+00  0.000000E+00       Funtag_D
      49  1.694729E+05  1.329573E+05       36515.6       24.9254      0.147910      0.861494      0.202840      0.228267    -0.0728708     0.0195070    0.00542096      0.876670       Opttag_D
      49       74.7360       1.73380       1.00000  0.000000E+00       1.00000  0.000000E+00      0.926717       64.2544       1.00000       38.9323  0.000000E+00       10.9991  0.000000E+00       10.9991  0.000000E+00  0.000000E+00      -21.9983       76.6492       2.58297        1       Scatag_D
      49       825.437       4188.94  0.000000E+00  0.000000E+00       2092.89  0.000000E+00       1884.85       2400.45  0.000000E+00       8889.44       33.4941       16200.1 -1.000000E+10 -1.000000E+10 -1.000000E+10 -1.000000E+10 -1.000000E+10  0.000000E+00  0.000000E+00  0.000000E+00  0.000000E+00  0.000000E+00  0.000000E+00  0.000000E+00  0.000000E+00  0.000000E+00  0.000000E+00  0.000000E+00  0.000000E+00       Funtag_D
      50  1.693098E+05  1.330042E+05       36305.6       28.8402      0.149469      0.864809      0.202957      0.228493    -0.0732807     0.0190265    0.00545862      0.876293       Opttag_D
      50       74.7572       1.73678       1.00000  0.000000E+00       1.00000  0.000000E+00      0.926944       64.0648       1.00000       39.0069  0.000000E+00       11.0055  0.000000E+00       11.0055  0.000000E+00  0.000000E+00      -22.0109       76.6492       2.58297        1       Scatag_D
      50       836.948       4185.34  0.000000E+00  0.000000E+00       2091.52  0.000000E+00       1862.63       2356.23  0.000000E+00       8807.89       34.5844       16130.5 -1.000000E+10 -1.000000E+10 -1.000000E+10 -1.000000E+10 -1.000000E+10  0.000000E+00  0.000000E+00  0.000000E+00  0.000000E+00  0.000000E+00  0.000000E+00  0.000000E+00  0.000000E+00  0.000000E+00  0.000000E+00  0.000000E+00  0.000000E+00       Funtag_D
      51  1.691488E+05  1.330254E+05       36123.4       18.6519      0.150163      0.863432      0.203007      0.228597    -0.0734656     0.0187611    0.00541843      0.873253       Opttag_D
      51       74.7677       1.73829       1.00000  0.000000E+00       1.00000  0.000000E+00      0.927052       63.9811       1.00000       39.0358  0.000000E+00       11.0076  0.000000E+00       11.0076  0.000000E+00  0.000000E+00      -22.0151       76.6492       2.58297        1       Scatag_D
      51       824.668       4156.35  0.000000E+00  0.000000E+00       2091.43  0.000000E+00       1852.74       2302.19  0.000000E+00       8768.60       35.4364       16091.9 -1.000000E+10 -1.000000E+10 -1.000000E+10 -1.000000E+10 -1.000000E+10  0.000000E+00  0.000000E+00  0.000000E+00  0.000000E+00  0.000000E+00  0.000000E+00  0.000000E+00  0.000000E+00  0.000000E+00  0.000000E+00  0.000000E+00  0.000000E+00       Funtag_D
      52  1.689225E+05  1.330673E+05       35855.2       20.4076      0.151656      0.855564      0.203102      0.228818    -0.0738307     0.0181756    0.00535854      0.870248       Opttag_D
      52       74.7923       1.74148       1.00000  0.000000E+00       1.00000  0.000000E+00      0.927292       63.8071       1.00000       39.0929  0.000000E+00       11.0110  0.000000E+00       11.0110  0.000000E+00  0.000000E+00      -22.0219       76.6492       2.58297        1       Scatag_D
      52       806.540       4127.79  0.000000E+00  0.000000E+00       2091.70  0.000000E+00       1834.76       2259.74  0.000000E+00       8683.36       36.8525       16014.5 -1.000000E+10 -1.000000E+10 -1.000000E+10 -1.000000E+10 -1.000000E+10  0.000000E+00  0.000000E+00  0.000000E+00  0.000000E+00  0.000000E+00  0.000000E+00  0.000000E+00  0.000000E+00  0.000000E+00  0.000000E+00  0.000000E+00  0.000000E+00       Funtag_D
      53  1.687688E+05  1.331016E+05       35667.2       27.6956      0.153156      0.849410      0.203184      0.229037    -0.0741301     0.0175908    0.00532786      0.867139       Opttag_D
      53       74.8191       1.74414       1.00000  0.000000E+00       1.00000  0.000000E+00      0.927522       63.6446       1.00000       39.1532  0.000000E+00       11.0127  0.000000E+00       11.0127  0.000000E+00  0.000000E+00      -22.0255       76.6492       2.58297        1       Scatag_D
      53       797.329       4098.35  0.000000E+00  0.000000E+00       2092.25  0.000000E+00       1823.68       2249.33  0.000000E+00       8605.96       35.5667       15964.8 -1.000000E+10 -1.000000E+10 -1.000000E+10 -1.000000E+10 -1.000000E+10  0.000000E+00  0.000000E+00  0.000000E+00  0.000000E+00  0.000000E+00  0.000000E+00  0.000000E+00  0.000000E+00  0.000000E+00  0.000000E+00  0.000000E+00  0.000000E+00       Funtag_D
      54  1.685759E+05  1.331359E+05       35440.0       20.3104      0.154781      0.844314      0.203265      0.229263    -0.0744295     0.0169935    0.00529672      0.863401       Opttag_D
      54       74.8492       1.74699       1.00000  0.000000E+00       1.00000  0.000000E+00      0.927765       63.4785       1.00000       39.2186  0.000000E+00       11.0142  0.000000E+00       11.0142  0.000000E+00  0.000000E+00      -22.0283       76.6492       2.58297        1       Scatag_D
      54       788.035       4063.10  0.000000E+00  0.000000E+00       2093.40  0.000000E+00       1814.74       2189.06  0.000000E+00       8528.88       33.5351       15929.2 -1.000000E+10 -1.000000E+10 -1.000000E+10 -1.000000E+10 -1.000000E+10  0.000000E+00  0.000000E+00  0.000000E+00  0.000000E+00  0.000000E+00  0.000000E+00  0.000000E+00  0.000000E+00  0.000000E+00  0.000000E+00  0.000000E+00  0.000000E+00       Funtag_D
      55  1.684188E+05  1.331912E+05       35227.6       31.7419      0.157417      0.843519      0.203394      0.229606    -0.0749125     0.0160381    0.00530531      0.861772       Opttag_D
      55       74.9007       1.75214       1.00000  0.000000E+00       1.00000  0.000000E+00      0.928152       63.2321       1.00000       39.3217  0.000000E+00       11.0164  0.000000E+00       11.0164  0.000000E+00  0.000000E+00      -22.0328       76.6492       2.58297        1       Scatag_D
      55       790.593       4047.78  0.000000E+00  0.000000E+00       2095.65  0.000000E+00       1799.24       2161.91  0.000000E+00       8415.72       31.9262       15884.7 -1.000000E+10 -1.000000E+10 -1.000000E+10 -1.000000E+10 -1.000000E+10  0.000000E+00  0.000000E+00  0.000000E+00  0.000000E+00  0.000000E+00  0.000000E+00  0.000000E+00  0.000000E+00  0.000000E+00  0.000000E+00  0.000000E+00  0.000000E+00       Funtag_D
      56  1.682914E+05  1.332036E+05       35087.8       16.4011      0.158088      0.848864      0.203421      0.229691    -0.0750199     0.0158093    0.00528746      0.858420       Opttag_D
      56       74.9149       1.75350       1.00000  0.000000E+00       1.00000  0.000000E+00      0.928251       63.1747       1.00000       39.3435  0.000000E+00       11.0172  0.000000E+00       11.0172  0.000000E+00  0.000000E+00      -22.0344       76.6492       2.58297        1       Scatag_D
      56       785.283       4016.35  0.000000E+00  0.000000E+00       2096.05  0.000000E+00       1791.93       2127.47  0.000000E+00       8387.54       32.2064       15851.0 -1.000000E+10 -1.000000E+10 -1.000000E+10 -1.000000E+10 -1.000000E+10  0.000000E+00  0.000000E+00  0.000000E+00  0.000000E+00  0.000000E+00  0.000000E+00  0.000000E+00  0.000000E+00  0.000000E+00  0.000000E+00  0.000000E+00  0.000000E+00       Funtag_D
      57  1.682176E+05  1.332041E+05       35013.5       14.4409      0.158237      0.855592      0.203417      0.229702    -0.0750245     0.0157649    0.00526105      0.857676       Opttag_D
      57       74.9193       1.75376       1.00000  0.000000E+00       1.00000  0.000000E+00      0.928276       63.1674       1.00000       39.3425  0.000000E+00       11.0180  0.000000E+00       11.0180  0.000000E+00  0.000000E+00      -22.0359       76.6492       2.58297        1       Scatag_D
      57       777.458       4009.39  0.000000E+00  0.000000E+00       2095.89  0.000000E+00       1786.11       2114.89  0.000000E+00       8379.11       33.0872       15817.6 -1.000000E+10 -1.000000E+10 -1.000000E+10 -1.000000E+10 -1.000000E+10  0.000000E+00  0.000000E+00  0.000000E+00  0.000000E+00  0.000000E+00  0.000000E+00  0.000000E+00  0.000000E+00  0.000000E+00  0.000000E+00  0.000000E+00  0.000000E+00       Funtag_D
      58  1.680541E+05  1.332243E+05       34829.7       18.0641      0.159489      0.882419      0.203456      0.229854    -0.0752012     0.0152682    0.00522005      0.856762       Opttag_D
      58       74.9488       1.75662       1.00000  0.000000E+00       1.00000  0.000000E+00      0.928460       63.0810       1.00000       39.3661  0.000000E+00       11.0202  0.000000E+00       11.0202  0.000000E+00  0.000000E+00      -22.0404       76.6492       2.58297        1       Scatag_D
      58       765.388       4000.85  0.000000E+00  0.000000E+00       2095.42  0.000000E+00       1765.08       2109.78  0.000000E+00       8325.11       34.9659       15733.2 -1.000000E+10 -1.000000E+10 -1.000000E+10 -1.000000E+10 -1.000000E+10  0.000000E+00  0.000000E+00  0.000000E+00  0.000000E+00  0.000000E+00  0.000000E+00  0.000000E+00  0.000000E+00  0.000000E+00  0.000000E+00  0.000000E+00  0.000000E+00       Funtag_D
      59  1.680146E+05  1.332851E+05       34729.5       44.2425      0.162424      0.914708      0.203589      0.230213    -0.0757313     0.0141967    0.00525957      0.860131       Opttag_D
      59       75.0114       1.76386       1.00000  0.000000E+00       1.00000  0.000000E+00      0.928843       62.8651       1.00000       39.4567  0.000000E+00       11.0211  0.000000E+00       11.0211  0.000000E+00  0.000000E+00      -22.0422       76.6492       2.58297        1       Scatag_D
      59       777.022       4032.38  0.000000E+00  0.000000E+00       2096.03  0.000000E+00       1742.35       2135.22  0.000000E+00       8222.19       33.5471       15690.8 -1.000000E+10 -1.000000E+10 -1.000000E+10 -1.000000E+10 -1.000000E+10  0.000000E+00  0.000000E+00  0.000000E+00  0.000000E+00  0.000000E+00  0.000000E+00  0.000000E+00  0.000000E+00  0.000000E+00  0.000000E+00  0.000000E+00  0.000000E+00       Funtag_D
      60  1.678639E+05  1.332811E+05       34582.9       16.8555      0.162330      0.913699      0.203583      0.230203    -0.0756961     0.0141874    0.00520609      0.854818       Opttag_D
      60       75.0096       1.76344       1.00000  0.000000E+00       1.00000  0.000000E+00      0.928830       62.8722       1.00000       39.4525  0.000000E+00       11.0207  0.000000E+00       11.0207  0.000000E+00  0.000000E+00      -22.0413       76.6492       2.58297        1       Scatag_D
      60       761.298       3982.71  0.000000E+00  0.000000E+00       2095.82  0.000000E+00       1742.65       2061.69  0.000000E+00       8224.06       33.3071       15681.3 -1.000000E+10 -1.000000E+10 -1.000000E+10 -1.000000E+10 -1.000000E+10  0.000000E+00  0.000000E+00  0.000000E+00  0.000000E+00  0.000000E+00  0.000000E+00  0.000000E+00  0.000000E+00  0.000000E+00  0.000000E+00  0.000000E+00  0.000000E+00       Funtag_D
      61  1.677868E+05  1.332988E+05       34488.0       10.7037      0.163218      0.919483      0.203625      0.230315    -0.0758511     0.0138180    0.00518673      0.853279       Opttag_D
      61       75.0283       1.76558       1.00000  0.000000E+00       1.00000  0.000000E+00      0.928938       62.8079       1.00000       39.4820  0.000000E+00       11.0197  0.000000E+00       11.0197  0.000000E+00  0.000000E+00      -22.0394       76.6492       2.58297        1       Scatag_D
      61       755.648       3968.39  0.000000E+00  0.000000E+00       2095.92  0.000000E+00       1737.57       2039.28  0.000000E+00       8194.78       32.3148       15664.1 -1.000000E+10 -1.000000E+10 -1.000000E+10 -1.000000E+10 -1.000000E+10  0.000000E+00  0.000000E+00  0.000000E+00  0.000000E+00  0.000000E+00  0.000000E+00  0.000000E+00  0.000000E+00  0.000000E+00  0.000000E+00  0.000000E+00  0.000000E+00       Funtag_D
      62  1.677077E+05  1.333361E+05       34371.6       14.2293      0.165022      0.931663      0.203712      0.230525    -0.0761762     0.0131145    0.00517617      0.851847       Opttag_D
      62       75.0667       1.77016       1.00000  0.000000E+00       1.00000  0.000000E+00      0.929151       62.6826       1.00000       39.5418  0.000000E+00       11.0181  0.000000E+00       11.0181  0.000000E+00  0.000000E+00      -22.0361       76.6492       2.58297        1       Scatag_D
      62       752.574       3955.08  0.000000E+00  0.000000E+00       2096.41  0.000000E+00       1727.33       2030.27  0.000000E+00       8139.20       31.3518       15639.4 -1.000000E+10 -1.000000E+10 -1.000000E+10 -1.000000E+10 -1.000000E+10  0.000000E+00  0.000000E+00  0.000000E+00  0.000000E+00  0.000000E+00  0.000000E+00  0.000000E+00  0.000000E+00  0.000000E+00  0.000000E+00  0.000000E+00  0.000000E+00       Funtag_D
      63  1.675949E+05  1.333858E+05       34209.1       22.7134      0.167550      0.941994      0.203831      0.230803    -0.0766102     0.0121548    0.00515047      0.850247       Opttag_D
      63       75.1225       1.77668       1.00000  0.000000E+00       1.00000  0.000000E+00      0.929431       62.5206       1.00000       39.6226  0.000000E+00       11.0151  0.000000E+00       11.0151  0.000000E+00  0.000000E+00      -22.0301       76.6492       2.58297        1       Scatag_D
      63       745.120       3940.24  0.000000E+00  0.000000E+00       2097.21  0.000000E+00       1713.26       2024.77  0.000000E+00       8066.32       31.6072       15590.6 -1.000000E+10 -1.000000E+10 -1.000000E+10 -1.000000E+10 -1.000000E+10  0.000000E+00  0.000000E+00  0.000000E+00  0.000000E+00  0.000000E+00  0.000000E+00  0.000000E+00  0.000000E+00  0.000000E+00  0.000000E+00  0.000000E+00  0.000000E+00       Funtag_D
      64  1.674692E+05  1.334756E+05       33993.5       19.8514      0.171720      0.954971      0.204053      0.231269    -0.0773938     0.0105853    0.00513446      0.846820       Opttag_D
      64       75.2144       1.78847       1.00000  0.000000E+00       1.00000  0.000000E+00      0.929869       62.2632       1.00000       39.7644  0.000000E+00       11.0096  0.000000E+00       11.0096  0.000000E+00  0.000000E+00      -22.0192       76.6492       2.58297        1       Scatag_D
      64       740.495       3908.54  0.000000E+00  0.000000E+00       2099.47  0.000000E+00       1694.58       2015.18  0.000000E+00       7962.22       31.8625       15541.2 -1.000000E+10 -1.000000E+10 -1.000000E+10 -1.000000E+10 -1.000000E+10  0.000000E+00  0.000000E+00  0.000000E+00  0.000000E+00  0.000000E+00  0.000000E+00  0.000000E+00  0.000000E+00  0.000000E+00  0.000000E+00  0.000000E+00  0.000000E+00       Funtag_D
      65  1.673635E+05  1.334867E+05       33876.7       12.9345      0.172567      0.949013      0.204082      0.231344    -0.0774907     0.0102997    0.00509972      0.845338       Opttag_D
      65       75.2356       1.79041       1.00000  0.000000E+00       1.00000  0.000000E+00      0.929943       62.2202       1.00000       39.7863  0.000000E+00       11.0085  0.000000E+00       11.0085  0.000000E+00  0.000000E+00      -22.0169       76.6492       2.58297        1       Scatag_D
      65       730.509       3894.87  0.000000E+00  0.000000E+00       2099.85  0.000000E+00       1690.00       1986.78  0.000000E+00       7939.57       31.8022       15503.4 -1.000000E+10 -1.000000E+10 -1.000000E+10 -1.000000E+10 -1.000000E+10  0.000000E+00  0.000000E+00  0.000000E+00  0.000000E+00  0.000000E+00  0.000000E+00  0.000000E+00  0.000000E+00  0.000000E+00  0.000000E+00  0.000000E+00  0.000000E+00       Funtag_D
      66  1.672478E+05  1.334885E+05       33759.3       16.3368      0.173388      0.933576      0.204093      0.231405    -0.0775063    0.00999413    0.00505783      0.844359       Opttag_D
      66       75.2588       1.79161       1.00000  0.000000E+00       1.00000  0.000000E+00      0.930002       62.1851       1.00000       39.7994  0.000000E+00       11.0073  0.000000E+00       11.0073  0.000000E+00  0.000000E+00      -22.0146       76.6492       2.58297        1       Scatag_D
      66       718.557       3885.85  0.000000E+00  0.000000E+00       2100.08  0.000000E+00       1684.29       1975.28  0.000000E+00       7915.36       31.6965       15448.2 -1.000000E+10 -1.000000E+10 -1.000000E+10 -1.000000E+10 -1.000000E+10  0.000000E+00  0.000000E+00  0.000000E+00  0.000000E+00  0.000000E+00  0.000000E+00  0.000000E+00  0.000000E+00  0.000000E+00  0.000000E+00  0.000000E+00  0.000000E+00       Funtag_D
      67  1.671628E+05  1.335037E+05       33659.1       19.3505      0.174791      0.917438      0.204137      0.231544    -0.0776385    0.00942065    0.00502739      0.843473       Opttag_D
      67       75.2952       1.79468       1.00000  0.000000E+00       1.00000  0.000000E+00      0.930116       62.1178       1.00000       39.8313  0.000000E+00       11.0052  0.000000E+00       11.0052  0.000000E+00  0.000000E+00      -22.0105       76.6492       2.58297        1       Scatag_D
      67       709.933       3877.70  0.000000E+00  0.000000E+00       2100.82  0.000000E+00       1676.54       1977.53  0.000000E+00       7880.36       31.5942       15404.6 -1.000000E+10 -1.000000E+10 -1.000000E+10 -1.000000E+10 -1.000000E+10  0.000000E+00  0.000000E+00  0.000000E+00  0.000000E+00  0.000000E+00  0.000000E+00  0.000000E+00  0.000000E+00  0.000000E+00  0.000000E+00  0.000000E+00  0.000000E+00       Funtag_D
      68  1.670646E+05  1.335170E+05       33547.6       12.7420      0.175966      0.905208      0.204172      0.231674    -0.0777549    0.00890110    0.00499916      0.841960       Opttag_D
      68       75.3256       1.79734       1.00000  0.000000E+00       1.00000  0.000000E+00      0.930212       62.0605       1.00000       39.8586  0.000000E+00       11.0029  0.000000E+00       11.0029  0.000000E+00  0.000000E+00      -22.0058       76.6492       2.58297        1       Scatag_D
      68       701.982       3863.80  0.000000E+00  0.000000E+00       2101.49  0.000000E+00       1670.48       1951.26  0.000000E+00       7853.16       31.1583       15374.3 -1.000000E+10 -1.000000E+10 -1.000000E+10 -1.000000E+10 -1.000000E+10  0.000000E+00  0.000000E+00  0.000000E+00  0.000000E+00  0.000000E+00  0.000000E+00  0.000000E+00  0.000000E+00  0.000000E+00  0.000000E+00  0.000000E+00  0.000000E+00       Funtag_D
      69  1.669567E+05  1.335391E+05       33417.6       13.8193      0.177544      0.894070      0.204227      0.231868    -0.0779476    0.00815061    0.00497448      0.839803       Opttag_D
      69       75.3655       1.80133       1.00000  0.000000E+00       1.00000  0.000000E+00      0.930339       61.9827       1.00000       39.8987  0.000000E+00       10.9992  0.000000E+00       10.9992  0.000000E+00  0.000000E+00      -21.9984       76.6492       2.58297        1       Scatag_D
      69       695.070       3844.03  0.000000E+00  0.000000E+00       2102.43  0.000000E+00       1662.99       1930.21  0.000000E+00       7819.42       30.5138       15332.9 -1.000000E+10 -1.000000E+10 -1.000000E+10 -1.000000E+10 -1.000000E+10  0.000000E+00  0.000000E+00  0.000000E+00  0.000000E+00  0.000000E+00  0.000000E+00  0.000000E+00  0.000000E+00  0.000000E+00  0.000000E+00  0.000000E+00  0.000000E+00       Funtag_D
      70  1.668621E+05  1.335693E+05       33292.8       19.9838      0.179574      0.906133      0.204300      0.232126    -0.0782110    0.00716013    0.00495959      0.837092       Opttag_D
      70       75.4171       1.80667       1.00000  0.000000E+00       1.00000  0.000000E+00      0.930497       61.8831       1.00000       39.9522  0.000000E+00       10.9942  0.000000E+00       10.9942  0.000000E+00  0.000000E+00      -21.9884       76.6492       2.58297        1       Scatag_D
      70       690.915       3819.26  0.000000E+00  0.000000E+00       2103.59  0.000000E+00       1655.26       1932.31  0.000000E+00       7778.98       30.0053       15282.5 -1.000000E+10 -1.000000E+10 -1.000000E+10 -1.000000E+10 -1.000000E+10  0.000000E+00  0.000000E+00  0.000000E+00  0.000000E+00  0.000000E+00  0.000000E+00  0.000000E+00  0.000000E+00  0.000000E+00  0.000000E+00  0.000000E+00  0.000000E+00       Funtag_D
      71  1.667714E+05  1.335928E+05       33178.6       13.9767      0.181070      0.916068      0.204358      0.232322    -0.0784163    0.00644452    0.00495157      0.834569       Opttag_D
      71       75.4558       1.81082       1.00000  0.000000E+00       1.00000  0.000000E+00      0.930605       61.8117       1.00000       39.9921  0.000000E+00       10.9903  0.000000E+00       10.9903  0.000000E+00  0.000000E+00      -21.9807       76.6492       2.58297        1       Scatag_D
      71       688.680       3796.26  0.000000E+00  0.000000E+00       2104.33  0.000000E+00       1650.88       1911.33  0.000000E+00       7750.79       29.8416       15246.4 -1.000000E+10 -1.000000E+10 -1.000000E+10 -1.000000E+10 -1.000000E+10  0.000000E+00  0.000000E+00  0.000000E+00  0.000000E+00  0.000000E+00  0.000000E+00  0.000000E+00  0.000000E+00  0.000000E+00  0.000000E+00  0.000000E+00  0.000000E+00       Funtag_D
      72  1.666949E+05  1.336047E+05       33090.2       11.7419      0.181881      0.921487      0.204387      0.232438    -0.0785202    0.00601840    0.00493753      0.832833       Opttag_D
      72       75.4779       1.81294       1.00000  0.000000E+00       1.00000  0.000000E+00      0.930658       61.7705       1.00000       40.0133  0.000000E+00       10.9882  0.000000E+00       10.9882  0.000000E+00  0.000000E+00      -21.9763       76.6492       2.58297        1       Scatag_D
      72       684.782       3780.49  0.000000E+00  0.000000E+00       2104.46  0.000000E+00       1648.87       1892.98  0.000000E+00       7734.81       29.8917       15213.9 -1.000000E+10 -1.000000E+10 -1.000000E+10 -1.000000E+10 -1.000000E+10  0.000000E+00  0.000000E+00  0.000000E+00  0.000000E+00  0.000000E+00  0.000000E+00  0.000000E+00  0.000000E+00  0.000000E+00  0.000000E+00  0.000000E+00  0.000000E+00       Funtag_D
      73  1.666130E+05  1.336253E+05       32987.7       15.3803      0.183282      0.930695      0.204435      0.232623    -0.0786996    0.00535655    0.00492455      0.831288       Opttag_D
      73       75.5164       1.81664       1.00000  0.000000E+00       1.00000  0.000000E+00      0.930742       61.6993       1.00000       40.0515  0.000000E+00       10.9848  0.000000E+00       10.9848  0.000000E+00  0.000000E+00      -21.9695       76.6492       2.58297        1       Scatag_D
      73       681.186       3766.47  0.000000E+00  0.000000E+00       2104.71  0.000000E+00       1646.13       1880.43  0.000000E+00       7708.61       29.7963       15170.4 -1.000000E+10 -1.000000E+10 -1.000000E+10 -1.000000E+10 -1.000000E+10  0.000000E+00  0.000000E+00  0.000000E+00  0.000000E+00  0.000000E+00  0.000000E+00  0.000000E+00  0.000000E+00  0.000000E+00  0.000000E+00  0.000000E+00  0.000000E+00       Funtag_D
      74  1.665563E+05  1.336468E+05       32909.4       15.6618      0.184597      0.939274      0.204486      0.232803    -0.0788877    0.00471032    0.00491702      0.830790       Opttag_D
      74       75.5524       1.82023       1.00000  0.000000E+00       1.00000  0.000000E+00      0.930816       61.6303       1.00000       40.0905  0.000000E+00       10.9818  0.000000E+00       10.9818  0.000000E+00  0.000000E+00      -21.9635       76.6492       2.58297        1       Scatag_D
      74       679.103       3761.96  0.000000E+00  0.000000E+00       2105.06  0.000000E+00       1642.46       1864.83  0.000000E+00       7685.59       29.5089       15140.9 -1.000000E+10 -1.000000E+10 -1.000000E+10 -1.000000E+10 -1.000000E+10  0.000000E+00  0.000000E+00  0.000000E+00  0.000000E+00  0.000000E+00  0.000000E+00  0.000000E+00  0.000000E+00  0.000000E+00  0.000000E+00  0.000000E+00  0.000000E+00       Funtag_D
      75  1.665043E+05  1.336535E+05       32850.9       10.6665      0.185034      0.942133      0.204502      0.232854    -0.0789454    0.00453865    0.00490092      0.830123       Opttag_D
      75       75.5648       1.82136       1.00000  0.000000E+00       1.00000  0.000000E+00      0.930837       61.6062       1.00000       40.1046  0.000000E+00       10.9810  0.000000E+00       10.9810  0.000000E+00  0.000000E+00      -21.9620       76.6492       2.58297        1       Scatag_D
      75       674.664       3755.92  0.000000E+00  0.000000E+00       2105.10  0.000000E+00       1638.77       1846.62  0.000000E+00       7676.98       29.2309       15123.6 -1.000000E+10 -1.000000E+10 -1.000000E+10 -1.000000E+10 -1.000000E+10  0.000000E+00  0.000000E+00  0.000000E+00  0.000000E+00  0.000000E+00  0.000000E+00  0.000000E+00  0.000000E+00  0.000000E+00  0.000000E+00  0.000000E+00  0.000000E+00       Funtag_D
      76  1.664256E+05  1.336788E+05       32746.8       11.2142      0.186500      0.951538      0.204561      0.233021    -0.0791668    0.00396552    0.00488447      0.829289       Opttag_D
      76       75.6053       1.82560       1.00000  0.000000E+00       1.00000  0.000000E+00      0.930911       61.5303       1.00000       40.1524  0.000000E+00       10.9781  0.000000E+00       10.9781  0.000000E+00  0.000000E+00      -21.9562       76.6492       2.58297        1       Scatag_D
      76       670.142       3748.38  0.000000E+00  0.000000E+00       2105.50  0.000000E+00       1627.65       1830.54  0.000000E+00       7650.72       28.7655       15085.1 -1.000000E+10 -1.000000E+10 -1.000000E+10 -1.000000E+10 -1.000000E+10  0.000000E+00  0.000000E+00  0.000000E+00  0.000000E+00  0.000000E+00  0.000000E+00  0.000000E+00  0.000000E+00  0.000000E+00  0.000000E+00  0.000000E+00  0.000000E+00       Funtag_D
      77  1.663777E+05  1.337129E+05       32664.8       19.1477      0.188329      0.962971      0.204642      0.233245    -0.0794641    0.00323175    0.00488630      0.828960       Opttag_D
      77       75.6560       1.83127       1.00000  0.000000E+00       1.00000  0.000000E+00      0.931001       61.4384       1.00000       40.2129  0.000000E+00       10.9743  0.000000E+00       10.9743  0.000000E+00  0.000000E+00      -21.9485       76.6492       2.58297        1       Scatag_D
      77       670.646       3745.41  0.000000E+00  0.000000E+00       2106.04  0.000000E+00       1616.26       1825.20  0.000000E+00       7619.90       28.8545       15052.5 -1.000000E+10 -1.000000E+10 -1.000000E+10 -1.000000E+10 -1.000000E+10  0.000000E+00  0.000000E+00  0.000000E+00  0.000000E+00  0.000000E+00  0.000000E+00  0.000000E+00  0.000000E+00  0.000000E+00  0.000000E+00  0.000000E+00  0.000000E+00       Funtag_D
      78  1.663169E+05  1.337347E+05       32582.3       11.9792      0.189414      0.969604      0.204694      0.233381    -0.0796539    0.00279180    0.00488010      0.828247       Opttag_D
      78       75.6863       1.83487       1.00000  0.000000E+00       1.00000  0.000000E+00      0.931054       61.3852       1.00000       40.2496  0.000000E+00       10.9719  0.000000E+00       10.9719  0.000000E+00  0.000000E+00      -21.9437       76.6492       2.58297        1       Scatag_D
      78       668.943       3738.96  0.000000E+00  0.000000E+00       2106.37  0.000000E+00       1609.02       1795.49  0.000000E+00       7601.81       29.0115       15032.7 -1.000000E+10 -1.000000E+10 -1.000000E+10 -1.000000E+10 -1.000000E+10  0.000000E+00  0.000000E+00  0.000000E+00  0.000000E+00  0.000000E+00  0.000000E+00  0.000000E+00  0.000000E+00  0.000000E+00  0.000000E+00  0.000000E+00  0.000000E+00       Funtag_D
      79  1.662384E+05  1.337780E+05       32460.4       10.0543      0.191480      0.981922      0.204791      0.233650    -0.0800318    0.00192954    0.00487546      0.827496       Opttag_D
      79       75.7449       1.84211       1.00000  0.000000E+00       1.00000  0.000000E+00      0.931153       61.2873       1.00000       40.3196  0.000000E+00       10.9672  0.000000E+00       10.9672  0.000000E+00  0.000000E+00      -21.9344       76.6492       2.58297        1       Scatag_D
      79       667.673       3732.19  0.000000E+00  0.000000E+00       2106.93  0.000000E+00       1596.28       1764.88  0.000000E+00       7568.00       29.2764       14995.2 -1.000000E+10 -1.000000E+10 -1.000000E+10 -1.000000E+10 -1.000000E+10  0.000000E+00  0.000000E+00  0.000000E+00  0.000000E+00  0.000000E+00  0.000000E+00  0.000000E+00  0.000000E+00  0.000000E+00  0.000000E+00  0.000000E+00  0.000000E+00       Funtag_D
      80  1.661938E+05  1.338106E+05       32383.2       13.8486      0.192993      0.990682      0.204863      0.233857    -0.0803168    0.00125757    0.00487545      0.826968       Opttag_D
      80       75.7887       1.84770       1.00000  0.000000E+00       1.00000  0.000000E+00      0.931224       61.2199       1.00000       40.3703  0.000000E+00       10.9636  0.000000E+00       10.9636  0.000000E+00  0.000000E+00      -21.9272       76.6492       2.58297        1       Scatag_D
      80       667.670       3727.42  0.000000E+00  0.000000E+00       2107.20  0.000000E+00       1588.21       1752.17  0.000000E+00       7543.92       29.0945       14967.5 -1.000000E+10 -1.000000E+10 -1.000000E+10 -1.000000E+10 -1.000000E+10  0.000000E+00  0.000000E+00  0.000000E+00  0.000000E+00  0.000000E+00  0.000000E+00  0.000000E+00  0.000000E+00  0.000000E+00  0.000000E+00  0.000000E+00  0.000000E+00       Funtag_D
      81  1.661358E+05  1.338473E+05       32288.5       10.8726      0.194675       1.00024      0.204943      0.234098    -0.0806365  4.940640E-04    0.00488021      0.826351       Opttag_D
      81       75.8381       1.85409       1.00000  0.000000E+00       1.00000  0.000000E+00      0.931302       61.1475       1.00000       40.4266  0.000000E+00       10.9596  0.000000E+00       10.9596  0.000000E+00  0.000000E+00      -21.9192       76.6492       2.58297        1       Scatag_D
      81       668.975       3721.87  0.000000E+00  0.000000E+00       2107.48  0.000000E+00       1579.98       1724.78  0.000000E+00       7518.31       28.7564       14938.4 -1.000000E+10 -1.000000E+10 -1.000000E+10 -1.000000E+10 -1.000000E+10  0.000000E+00  0.000000E+00  0.000000E+00  0.000000E+00  0.000000E+00  0.000000E+00  0.000000E+00  0.000000E+00  0.000000E+00  0.000000E+00  0.000000E+00  0.000000E+00       Funtag_D
      82  1.660732E+05  1.338979E+05       32175.2       17.4226      0.197066       1.01357      0.205047      0.234454    -0.0810786 -5.750225E-04    0.00489246      0.825450       Opttag_D
      82       75.9107       1.86358       1.00000  0.000000E+00       1.00000  0.000000E+00      0.931409       61.0594       1.00000       40.5024  0.000000E+00       10.9536  0.000000E+00       10.9536  0.000000E+00  0.000000E+00      -21.9072       76.6492       2.58297        1       Scatag_D
      82       672.336       3713.76  0.000000E+00  0.000000E+00       2107.59  0.000000E+00       1568.17       1706.42  0.000000E+00       7483.16       28.3360       14895.5 -1.000000E+10 -1.000000E+10 -1.000000E+10 -1.000000E+10 -1.000000E+10  0.000000E+00  0.000000E+00  0.000000E+00  0.000000E+00  0.000000E+00  0.000000E+00  0.000000E+00  0.000000E+00  0.000000E+00  0.000000E+00  0.000000E+00  0.000000E+00       Funtag_D
      83  1.660115E+05  1.339216E+05       32090.0       10.7489      0.198291       1.02041      0.205093      0.234641    -0.0812848   -0.00113134    0.00489385      0.824377       Opttag_D
      83       75.9492       1.86839       1.00000  0.000000E+00       1.00000  0.000000E+00      0.931465       61.0191       1.00000       40.5393  0.000000E+00       10.9507  0.000000E+00       10.9507  0.000000E+00  0.000000E+00      -21.9014       76.6492       2.58297        1       Scatag_D
      83       672.718       3704.11  0.000000E+00  0.000000E+00       2107.50  0.000000E+00       1561.62       1678.65  0.000000E+00       7465.52       28.1921       14871.6 -1.000000E+10 -1.000000E+10 -1.000000E+10 -1.000000E+10 -1.000000E+10  0.000000E+00  0.000000E+00  0.000000E+00  0.000000E+00  0.000000E+00  0.000000E+00  0.000000E+00  0.000000E+00  0.000000E+00  0.000000E+00  0.000000E+00  0.000000E+00       Funtag_D
      84  1.659685E+05  1.339233E+05       32045.1       9.98492      0.198610       1.02240      0.205090      0.234679    -0.0813003   -0.00123661    0.00488332      0.823688       Opttag_D
      84       75.9610       1.86951       1.00000  0.000000E+00       1.00000  0.000000E+00      0.931478       61.0180       1.00000       40.5432  0.000000E+00       10.9499  0.000000E+00       10.9499  0.000000E+00  0.000000E+00      -21.8999       76.6492       2.58297        1       Scatag_D
      84       669.827       3697.92  0.000000E+00  0.000000E+00       2107.11  0.000000E+00       1558.19       1669.62  0.000000E+00       7459.37       28.2964       14854.8 -1.000000E+10 -1.000000E+10 -1.000000E+10 -1.000000E+10 -1.000000E+10  0.000000E+00  0.000000E+00  0.000000E+00  0.000000E+00  0.000000E+00  0.000000E+00  0.000000E+00  0.000000E+00  0.000000E+00  0.000000E+00  0.000000E+00  0.000000E+00       Funtag_D
      85  1.658994E+05  1.339437E+05       31955.7       18.4329      0.200267       1.05640      0.205112      0.234875    -0.0814783   -0.00179925    0.00487526      0.822542       Opttag_D
      85       76.0171       1.87573       1.00000  0.000000E+00       1.00000  0.000000E+00      0.931551       60.9961       1.00000       40.5777  0.000000E+00       10.9465  0.000000E+00       10.9465  0.000000E+00  0.000000E+00      -21.8930       76.6492       2.58297        1       Scatag_D
      85       667.617       3687.63  0.000000E+00  0.000000E+00       2106.13  0.000000E+00       1544.90       1673.43  0.000000E+00       7435.32       28.5962       14812.1 -1.000000E+10 -1.000000E+10 -1.000000E+10 -1.000000E+10 -1.000000E+10  0.000000E+00  0.000000E+00  0.000000E+00  0.000000E+00  0.000000E+00  0.000000E+00  0.000000E+00  0.000000E+00  0.000000E+00  0.000000E+00  0.000000E+00  0.000000E+00       Funtag_D
      86  1.658430E+05  1.339771E+05       31865.9       14.5680      0.202109       1.10017      0.205167      0.235103    -0.0817692   -0.00245857    0.00487798      0.822095       Opttag_D
      86       76.0750       1.88326       1.00000  0.000000E+00       1.00000  0.000000E+00      0.931634       60.9587       1.00000       40.6277  0.000000E+00       10.9419  0.000000E+00       10.9419  0.000000E+00  0.000000E+00      -21.8838       76.6492       2.58297        1       Scatag_D
      86       668.362       3683.63  0.000000E+00  0.000000E+00       2106.04  0.000000E+00       1533.57       1644.69  0.000000E+00       7414.21       28.5667       14786.8 -1.000000E+10 -1.000000E+10 -1.000000E+10 -1.000000E+10 -1.000000E+10  0.000000E+00  0.000000E+00  0.000000E+00  0.000000E+00  0.000000E+00  0.000000E+00  0.000000E+00  0.000000E+00  0.000000E+00  0.000000E+00  0.000000E+00  0.000000E+00       Funtag_D
      87  1.658057E+05  1.339828E+05       31823.0       9.28281      0.202395       1.10440      0.205179      0.235139    -0.0818186   -0.00255887    0.00486524      0.821703       Opttag_D
      87       76.0835       1.88440       1.00000  0.000000E+00       1.00000  0.000000E+00      0.931650       60.9512       1.00000       40.6367  0.000000E+00       10.9413  0.000000E+00       10.9413  0.000000E+00  0.000000E+00      -21.8826       76.6492       2.58297        1       Scatag_D
      87       664.877       3680.12  0.000000E+00  0.000000E+00       2106.04  0.000000E+00       1530.97       1626.80  0.000000E+00       7411.03       28.4607       14774.7 -1.000000E+10 -1.000000E+10 -1.000000E+10 -1.000000E+10 -1.000000E+10  0.000000E+00  0.000000E+00  0.000000E+00  0.000000E+00  0.000000E+00  0.000000E+00  0.000000E+00  0.000000E+00  0.000000E+00  0.000000E+00  0.000000E+00  0.000000E+00       Funtag_D
      88  1.657504E+05  1.340151E+05       31735.3       7.89247      0.204068       1.13687      0.205238      0.235371    -0.0821011   -0.00319416    0.00485276      0.821182       Opttag_D
      88       76.1351       1.89131       1.00000  0.000000E+00       1.00000  0.000000E+00      0.931735       60.9143       1.00000       40.6865  0.000000E+00       10.9374  0.000000E+00       10.9374  0.000000E+00  0.000000E+00      -21.8749       76.6492       2.58297        1       Scatag_D
      88       661.470       3675.45  0.000000E+00  0.000000E+00       2105.99  0.000000E+00       1520.56       1609.94  0.000000E+00       7394.74       28.2586       14738.9 -1.000000E+10 -1.000000E+10 -1.000000E+10 -1.000000E+10 -1.000000E+10  0.000000E+00  0.000000E+00  0.000000E+00  0.000000E+00  0.000000E+00  0.000000E+00  0.000000E+00  0.000000E+00  0.000000E+00  0.000000E+00  0.000000E+00  0.000000E+00       Funtag_D
      89  1.657140E+05  1.340485E+05       31665.5       12.2217      0.205949       1.17766      0.205296      0.235640    -0.0823919   -0.00391655    0.00484474      0.820610       Opttag_D
      89       76.1946       1.89910       1.00000  0.000000E+00       1.00000  0.000000E+00      0.931833       60.8811       1.00000       40.7402  0.000000E+00       10.9324  0.000000E+00       10.9324  0.000000E+00  0.000000E+00      -21.8648       76.6492       2.58297        1       Scatag_D
      89       659.285       3670.33  0.000000E+00  0.000000E+00       2105.77  0.000000E+00       1511.21       1604.53  0.000000E+00       7378.68       28.1727       14707.5 -1.000000E+10 -1.000000E+10 -1.000000E+10 -1.000000E+10 -1.000000E+10  0.000000E+00  0.000000E+00  0.000000E+00  0.000000E+00  0.000000E+00  0.000000E+00  0.000000E+00  0.000000E+00  0.000000E+00  0.000000E+00  0.000000E+00  0.000000E+00       Funtag_D
      90  1.656648E+05  1.340867E+05       31578.1       9.34724      0.208139       1.22426      0.205359      0.235949    -0.0827253   -0.00476960    0.00483335      0.819942       Opttag_D
      90       76.2643       1.90827       1.00000  0.000000E+00       1.00000  0.000000E+00      0.931947       60.8491       1.00000       40.8029  0.000000E+00       10.9263  0.000000E+00       10.9263  0.000000E+00  0.000000E+00      -21.8526       76.6492       2.58297        1       Scatag_D
      90       656.189       3664.36  0.000000E+00  0.000000E+00       2105.48  0.000000E+00       1502.05       1585.61  0.000000E+00       7362.98       28.1017       14673.3 -1.000000E+10 -1.000000E+10 -1.000000E+10 -1.000000E+10 -1.000000E+10  0.000000E+00  0.000000E+00  0.000000E+00  0.000000E+00  0.000000E+00  0.000000E+00  0.000000E+00  0.000000E+00  0.000000E+00  0.000000E+00  0.000000E+00  0.000000E+00       Funtag_D
      91  1.656064E+05  1.341225E+05       31483.9       13.8408      0.210764       1.27709      0.205410      0.236303    -0.0830383   -0.00574227    0.00481446      0.818652       Opttag_D
      91       76.3497       1.91866       1.00000  0.000000E+00       1.00000  0.000000E+00      0.932091       60.8282       1.00000       40.8730  0.000000E+00       10.9182  0.000000E+00       10.9182  0.000000E+00  0.000000E+00      -21.8363       76.6492       2.58297        1       Scatag_D
      91       651.068       3652.84  0.000000E+00  0.000000E+00       2104.67  0.000000E+00       1493.72       1583.24  0.000000E+00       7347.53       28.0480       14622.7 -1.000000E+10 -1.000000E+10 -1.000000E+10 -1.000000E+10 -1.000000E+10  0.000000E+00  0.000000E+00  0.000000E+00  0.000000E+00  0.000000E+00  0.000000E+00  0.000000E+00  0.000000E+00  0.000000E+00  0.000000E+00  0.000000E+00  0.000000E+00       Funtag_D
      92  1.655509E+05  1.341429E+05       31407.9       11.1536      0.212750       1.31457      0.205431      0.236552    -0.0832162   -0.00646259    0.00480339      0.817251       Opttag_D
      92       76.4154       1.92607       1.00000  0.000000E+00       1.00000  0.000000E+00      0.932205       60.8249       1.00000       40.9228  0.000000E+00       10.9112  0.000000E+00       10.9112  0.000000E+00  0.000000E+00      -21.8224       76.6492       2.58297        1       Scatag_D
      92       648.078       3640.35  0.000000E+00  0.000000E+00       2103.89  0.000000E+00       1489.31       1579.13  0.000000E+00       7338.21       28.0608       14580.9 -1.000000E+10 -1.000000E+10 -1.000000E+10 -1.000000E+10 -1.000000E+10  0.000000E+00  0.000000E+00  0.000000E+00  0.000000E+00  0.000000E+00  0.000000E+00  0.000000E+00  0.000000E+00  0.000000E+00  0.000000E+00  0.000000E+00  0.000000E+00       Funtag_D
      93  1.655041E+05  1.341301E+05       31374.0       9.50413      0.212983       1.31532      0.205392      0.236550    -0.0831040   -0.00647596    0.00479410      0.816152       Opttag_D
      93       76.4260       1.92573       1.00000  0.000000E+00       1.00000  0.000000E+00      0.932228       60.8425       1.00000       40.9186  0.000000E+00       10.9097  0.000000E+00       10.9097  0.000000E+00  0.000000E+00      -21.8194       76.6492       2.58297        1       Scatag_D
      93       645.575       3630.56  0.000000E+00  0.000000E+00       2103.03  0.000000E+00       1490.22       1581.24  0.000000E+00       7336.58       28.0085       14558.8 -1.000000E+10 -1.000000E+10 -1.000000E+10 -1.000000E+10 -1.000000E+10  0.000000E+00  0.000000E+00  0.000000E+00  0.000000E+00  0.000000E+00  0.000000E+00  0.000000E+00  0.000000E+00  0.000000E+00  0.000000E+00  0.000000E+00  0.000000E+00       Funtag_D
      94  1.654266E+05  1.340906E+05       31336.0       13.8187      0.213596       1.31407      0.205277      0.236524    -0.0827597   -0.00645617    0.00479923      0.813877       Opttag_D
      94       76.4546       1.92440       1.00000  0.000000E+00       1.00000  0.000000E+00      0.932296       60.8984       1.00000       40.9019  0.000000E+00       10.9060  0.000000E+00       10.9060  0.000000E+00  0.000000E+00      -21.8120       76.6492       2.58297        1       Scatag_D
      94       646.957       3610.35  0.000000E+00  0.000000E+00       2100.76  0.000000E+00       1493.47       1610.80  0.000000E+00       7332.88       27.6854       14513.1 -1.000000E+10 -1.000000E+10 -1.000000E+10 -1.000000E+10 -1.000000E+10  0.000000E+00  0.000000E+00  0.000000E+00  0.000000E+00  0.000000E+00  0.000000E+00  0.000000E+00  0.000000E+00  0.000000E+00  0.000000E+00  0.000000E+00  0.000000E+00       Funtag_D
      95  1.653786E+05  1.340979E+05       31280.7       20.4621      0.215792       1.34558      0.205260      0.236739    -0.0828236   -0.00706625    0.00480468      0.812752       Opttag_D
      95       76.5285       1.93137       1.00000  0.000000E+00       1.00000  0.000000E+00      0.932439       60.9210       1.00000       40.9472  0.000000E+00       10.8983  0.000000E+00       10.8983  0.000000E+00  0.000000E+00      -21.7965       76.6492       2.58297        1       Scatag_D
      95       648.427       3600.38  0.000000E+00  0.000000E+00       2099.89  0.000000E+00       1489.18       1630.78  0.000000E+00       7323.85       27.3617       14460.8 -1.000000E+10 -1.000000E+10 -1.000000E+10 -1.000000E+10 -1.000000E+10  0.000000E+00  0.000000E+00  0.000000E+00  0.000000E+00  0.000000E+00  0.000000E+00  0.000000E+00  0.000000E+00  0.000000E+00  0.000000E+00  0.000000E+00  0.000000E+00       Funtag_D
      96  1.653225E+05  1.340949E+05       31227.6       11.1245      0.215612       1.34124      0.205255      0.236712    -0.0827971   -0.00698665    0.00478992      0.812394       Opttag_D
      96       76.5230       1.93059       1.00000  0.000000E+00       1.00000  0.000000E+00      0.932432       60.9224       1.00000       40.9406  0.000000E+00       10.8993  0.000000E+00       10.8993  0.000000E+00  0.000000E+00      -21.7986       76.6492       2.58297        1       Scatag_D
      96       644.450       3597.20  0.000000E+00  0.000000E+00       2099.86  0.000000E+00       1487.63       1601.70  0.000000E+00       7322.47       27.3074       14447.0 -1.000000E+10 -1.000000E+10 -1.000000E+10 -1.000000E+10 -1.000000E+10  0.000000E+00  0.000000E+00  0.000000E+00  0.000000E+00  0.000000E+00  0.000000E+00  0.000000E+00  0.000000E+00  0.000000E+00  0.000000E+00  0.000000E+00  0.000000E+00       Funtag_D
      97  1.652655E+05  1.341036E+05       31161.9       8.37295      0.216684       1.35604      0.205259      0.236817    -0.0828735   -0.00729196    0.00478262      0.811924       Opttag_D
      97       76.5583       1.93443       1.00000  0.000000E+00       1.00000  0.000000E+00      0.932502       60.9293       1.00000       40.9649  0.000000E+00       10.8963  0.000000E+00       10.8963  0.000000E+00  0.000000E+00      -21.7925       76.6492       2.58297        1       Scatag_D
      97       642.487       3593.04  0.000000E+00  0.000000E+00       2099.74  0.000000E+00       1482.53       1597.19  0.000000E+00       7316.05       27.1935       14403.6 -1.000000E+10 -1.000000E+10 -1.000000E+10 -1.000000E+10 -1.000000E+10  0.000000E+00  0.000000E+00  0.000000E+00  0.000000E+00  0.000000E+00  0.000000E+00  0.000000E+00  0.000000E+00  0.000000E+00  0.000000E+00  0.000000E+00  0.000000E+00       Funtag_D
      98  1.652267E+05  1.341096E+05       31117.1       9.65576      0.217782       1.37048      0.205258      0.236914    -0.0829256   -0.00759086    0.00477748      0.811490       Opttag_D
      98       76.5952       1.93815       1.00000  0.000000E+00       1.00000  0.000000E+00      0.932572       60.9401       1.00000       40.9895  0.000000E+00       10.8925  0.000000E+00       10.8925  0.000000E+00  0.000000E+00      -21.7851       76.6492       2.58297        1       Scatag_D
      98       641.107       3589.21  0.000000E+00  0.000000E+00       2099.60  0.000000E+00       1479.37       1601.61  0.000000E+00       7310.86       27.1438       14368.2 -1.000000E+10 -1.000000E+10 -1.000000E+10 -1.000000E+10 -1.000000E+10  0.000000E+00  0.000000E+00  0.000000E+00  0.000000E+00  0.000000E+00  0.000000E+00  0.000000E+00  0.000000E+00  0.000000E+00  0.000000E+00  0.000000E+00  0.000000E+00       Funtag_D
      99  1.652042E+05  1.341085E+05       31095.6       28.0251      0.219653       1.38858      0.205229      0.237044    -0.0829163   -0.00798707    0.00476163      0.811263       Opttag_D
      99       76.6611       1.94348       1.00000  0.000000E+00       1.00000  0.000000E+00      0.932694       60.9694       1.00000       41.0273  0.000000E+00       10.8856  0.000000E+00       10.8856  0.000000E+00  0.000000E+00      -21.7711       76.6492       2.58297        1       Scatag_D
      99       636.859       3587.20  0.000000E+00  0.000000E+00       2099.34  0.000000E+00       1475.64       1661.06  0.000000E+00       7300.45       26.9178       14308.2 -1.000000E+10 -1.000000E+10 -1.000000E+10 -1.000000E+10 -1.000000E+10  0.000000E+00  0.000000E+00  0.000000E+00  0.000000E+00  0.000000E+00  0.000000E+00  0.000000E+00  0.000000E+00  0.000000E+00  0.000000E+00  0.000000E+00  0.000000E+00       Funtag_D

   MaxLik Scaling Cycle 0104

   Wilson plot

   Statistics in resolution bins after refinement cycle 0003
```


|  |  |
| --- | --- |
|  | - Rxpct factors plot- Rxpct factors table- <Log-Lik Gain>- Ln Sigma A- Correlation Coefficients |


```
   Iters     Tot_Funct    Xray_Funct    Geom_Funct          Grms     rmsD2init     maxD2init             R         Rfree           LLG       LLGfree     rms(bond)    rms(angle)
   Iters             K   B_IMPF_FRAG        K_MISS   B_TEMP_MISS   K_IMPF_MISS   B_IMPF_MISS        K_SOLV   B_TEMP_SOLV   K_IMPF_SOLV   B_IMPF_SOLV        B_TEMP     B_TEMP_11     B_TEMP_12     B_TEMP_22     B_TEMP_13     B_TEMP_23     B_TEMP_33      K_WILSON      B_WILSON      FGCYCLES
   Iters          BOND         ANGLE      IMPROPER       TORSION    SINTORSION      TRIGONAL        PLANAR  BCORRELATION       CONTACT     IDEALDIST        CHIRAL    SIMILARITY       OCCSSUM  UTILDISTANCE     UTILANGLE   UTILTORSION        RHOFIT        ADP-01        ADP-02        ADP-03        ADP-04        ADP-05        ADP-06        ADP-07        ADP-08        ADP-09        ADP-10      RHOFITPC            QM
     100  1.651269E+05  1.341055E+05       31021.4       8.75520      0.219716       1.38807      0.205223      0.237031    -0.0828899   -0.00796312    0.00474916      0.810599       Opttag_D
     100       76.6643       1.94345       1.00000  0.000000E+00       1.00000  0.000000E+00      0.932698       60.9731       1.00000       41.0277  0.000000E+00       10.8850  0.000000E+00       10.8850  0.000000E+00  0.000000E+00      -21.7701       76.6492       2.58297        1       Scatag_D
     100       633.528       3581.33  0.000000E+00  0.000000E+00       2099.22  0.000000E+00       1475.97       1603.18  0.000000E+00       7299.30       26.9393       14302.0 -1.000000E+10 -1.000000E+10 -1.000000E+10 -1.000000E+10 -1.000000E+10  0.000000E+00  0.000000E+00  0.000000E+00  0.000000E+00  0.000000E+00  0.000000E+00  0.000000E+00  0.000000E+00  0.000000E+00  0.000000E+00  0.000000E+00  0.000000E+00       Funtag_D
   Geometry Restraint Deviations SCREEN Information

 
 No -bcorrel_zeroocchydrogens_factor flag found - keeping default: will set B for OCC=0 hydrogens to    1.200 times heavy-atom B


 TLS information. Total number of groups:     2


 Group   Natoms    FixRB   FixTLS   Group Tag    Group Spec
     1     2770      Y        Y     tls1    { A|* }
     2     2770      Y        Y     tls2    { B|* }
 
NOTE BUSTER_TLS_SET tls1 { A|* }
NOTE BUSTER_TLS_T   tls1     -5.14088E-02  1.73416E-02 -8.39463E-02 -8.98636E-03  2.71161E-02 -1.44550E-02
NOTE BUSTER_TLS_L   tls1      5.75955E-01  6.03476E-01  7.20382E-02  4.29501E-01 -2.01688E-01 -7.44442E-02
NOTE BUSTER_TLS_S   tls1      2.27569E-01 -1.40005E-01  1.08103E-01 -1.53415E-01 -6.25968E-02 -9.66768E-02 -5.90336E-02  8.69448E-04
NOTE BUSTER_TLS_O   tls1     -2.34016E+01 -8.37990E+01  1.17779E+01
NOTE BUSTER_TLS_FIX tls1 ALL
 
NOTE BUSTER_TLS_SET tls2 { B|* }
NOTE BUSTER_TLS_T   tls2     -6.53873E-02 -2.61952E-02 -1.61508E-02  1.87470E-02  4.10405E-02  3.19663E-03
NOTE BUSTER_TLS_L   tls2      3.60326E-01  5.33835E-01  2.67053E-01  2.19756E-01 -1.98637E-01 -2.16915E-01
NOTE BUSTER_TLS_S   tls2     -1.51205E-02  2.91585E-02 -3.20999E-02 -6.51209E-03  1.30308E-01  8.58995E-03 -4.97491E-02  3.47416E-02
NOTE BUSTER_TLS_O   tls2      2.21707E+01 -7.20650E+01  3.37962E+00
NOTE BUSTER_TLS_FIX tls2 ALL

 For final position, Geometry function summary
     term                           number     weight       rms        GooF      function     fn/numb
     ================================================================================================
     BOND bond lengths (angs)       5655       2.000      0.0047       0.237       633.53       0.112
     ANGL bond angles (degs)        7674       2.000        0.81       0.483      3581.33       0.467
     TORS split-harmonic tors       1270       0.000       16.53       1.040         0.00       0.000
     SINTOR sinusoidal tors         1270       2.000       16.53                  2099.22       1.653
     PLAN general plane (angs)       963       5.000      0.0111       0.554      1475.97       1.533
     BCORrelations (angs^2)         5655      10.000       0.472       0.168      1603.18       0.283
     CONT (bad contacts)               0       5.000                                 0.00
     IDEAL(ideal-dist contact)      5211       4.000                              7299.30
     CHIRAL (gelly semiharmon)       740       5.000                                26.94
     SIM similarity restraint for NCS and/or TARGET                              14301.95
     OCCSUM restraint on sum of occupanies                                           0.00       as none defined
     DISTAN utility restraints interatomic distance                                  0.00       as none defined
     UTILANGLE utility angle restraints                                              0.00       as none defined
     UTILTOR utility restraints on torsion angles                                    0.00       as none defined
     Peptide omega torsion angles                           2.33 degs - monitor only
 
 WARNING: Have |delta/sigma| deviations > 5.0 sigma. Number of outliers for each term: 
 WARNING:         1 bond angles.  Worst is     5.0 sigs   134.22 degs B|28:C.A(ASN)=B|29:N(ASN)=B|29:CA(ASN)

 Weighted rms      0.0047         0.811  (temporary rms bond and angle for BUSTER to pick up!)
 Weighted intra-residual rms(bond) for StdProtein (excluding H)      0.0045
 Weighted intra-residual rms(bond) for Buffer     (excluding H)      0.0036
 Outputing geometry progress graph in plotmtv format to file:Geom_Res.mtv

 ############### Running final geometry/screen analysis (screen_final.txt)

 s/r gelly normal termination
```

Statistics and scale factors vs. cycle number
```

|  |  |  |
| --- | --- | --- |
|  | - Rxpct factors- <Log-Lik Gain>- GEOM Residuals- Ln Sigma_A_ | - Scale Factors- B Factors- Imperfection Factors |

```
   Final Geometry Information


---


### 2. Final results

Statistics and scale factors vs. cycle number
```

|  |  |  |
| --- | --- | --- |
|  | - Rxpct factors- <Log-Lik Gain>- GEOM Residuals- Ln Sigma_A_ | - Scale Factors- B Factors- Imperfection Factors |

```
   Average Figure of Merit
   Structure Factor Amplitudes


 # normal termination

  Program stopped.
```

closesok message: time spent in tree = 1230.98 (user) 32.42 (sys)

---

# Normal termination

```
 Resources usage summary:
     Time spent in client ...................  1230.99 (user)       32.44 (system)
     Time spent in scripts (    27 calls) ...     1.60 (user)        0.13 (system)
     Time spent in server ...................     0.00 (user)        0.00 (system)

     Max resident memory used (kb) .......... 445584
     Number of page faults .................. 0 (major)    384892 (minor)
     Number of context switches ............. 9958 (niced)    4508 (other)
```
